# Exosome-mediated secretion of miR-127-3p regulated by RAB27A accelerates metastasis in renal cell carcinoma

**DOI:** 10.1186/s12935-024-03334-0

**Published:** 2024-04-29

**Authors:** Dae Hyun Song, Jong Sil Lee, Jeong-Hee Lee, Dong Chul Kim, Jung Wook Yang, Min Hye Kim, Ji Min Na, Hyun-kyung Cho, Jiyun Yoo, Hyo Jung An

**Affiliations:** 1 Department of Pathology, Gyeongsang National University Changwon Hospital, Changwon, South Korea; 2 Institute of Medical Sciences, Gyeongsang National University, Jinju, South Korea; 3 Department of Pathology, Gyeongsang National University School of Medicine, Jinju, South Korea; 4 Department of Pathology, Gyeongsang National University Hospital, Jinju, South Korea; 5 Department of Ophthalmology, Gyeongsang National University Changwon Hospital, Gyeongsang National University, School of Medicine, Changwon, South Korea; 6 Division of Applied Life Science (BK21 Plus) and Research Institute of Life Sciences, Gyeongsang National University, Jinju, South Korea

## Abstract

**Background:**

The exosome-mediated extracellular secretion of miRNAs occurs in many cancers, and RAB27A is a potent regulator of exosome secretion. For metastatic renal cell carcinoma (RCC), this study examines the mechanisms of cancer metastasis via the RAB27A-regulated secretion of specific miRNAs.

**Methods:**

RAB27A knockdown (KD) and overexpressing (OE) RCC cells were used to examine cell migration and adhesion. The particle counts and sizes of exosomes in RAB27A OE cells were analyzed using Exoview, and those of intraluminal vesicles (ILV) and multivesicular bodies (MVB) were measured using an electron microscope. Analysis of RNA sequences, protein–protein interaction networks, and the competing endogenous RNA (ceRNA) network were used to identify representative downregulated miRNAs that are likely to undergo cargo-sorting into exosomes and subsequent secretion. A molecular beacon of miR-137-3p, one of the most representatively downregulated genes with a fold change of 339, was produced, and its secretion was analyzed using Exoview. RAB27A OE and control cells were incubated in an exosome-containing media to determine the uptake of tumor suppressor miRNAs that affect cancer cell metastasis.

**Results:**

Migration and cell adhesion were higher in RAB27A OE cells than in RAB27A KD cells. Electron microscopy revealed that the numbers of multivesicular bodies and intraluminal vesicles per cell were higher in RAB27A OE cells than in control cells, suggesting their secretion. The finding revealed that miR-127-3p was sorted into exosomes and disposed of extracellularly. Protein–protein interaction analysis revealed MYCN to be the most significant hub for RAB27A-OE RCC cells. ceRNA network analysis revealed that MAPK4 interacted strongly with miR-127-3p.

**Conclusion:**

The disposal of miR-127-3p through exosome secretion in RAB27A overexpressing cells may not inhibit the MAPK pathway to gain metastatic potential by activating MYCN. The exosomes containing miRNAs are valuable therapeutic targets for cancer treatment.

**Supplementary Information:**

The online version contains supplementary material available at 10.1186/s12935-024-03334-0.

## Background

Renal cell carcinoma accounts for 3.8% of all new cancers in the United States; approximately 70% are clear cell renal cell carcinoma (CCRCC), and 10–15% are papillary renal cell carcinomas. According to the American Cancer Society, the five-year survival rates for stages 1–4 are 81, 74, 53, and 8%, respectively. Supportive care remains the mainstay of therapy for patients with relapsed stage 4 disease and brain or bone metastases. Supportive care includes surgery, stereotactic radiotherapy, whole-brain irradiation for multiple brain metastases, and bone-modifying agents with bone metastasis. (National Comprehensive Cancer Network [NCCN] guidelines, version 4.2023). There is an urgent need to prevent and directly target cancer cell metastasis in RCC.

Cancer cell circulation and dissemination may initiate metastatic processes, which include invasion, dissemination into the blood or lymphatic system, survival during circulation, arrest in the tissue bed, extravasation, dormancy, and metastatic reawakening. [1] Nanoparticles, including exosomes, are components of the tumor microenvironment and are crucial factors involved in cancer metastasis. [2] Exosomes comprise a lipid bilayer, and their cargo contains genetic information, including RNA and miRNA, lipids, and proteins. Exosomes have a very similar structure to the cells of origin; intracellularly, they occur as small intraluminal vesicles (ILVs), but following secretion, they become extracellular vesicles (i.e., exosomes). [3–7] Exosomes maintain genetic functions and alter the behavior of cells by transferring their internal cargo. [3–6, 8] Exosome function is therefore being studied in various pathophysiological processes, including cancer, immune responses, and cardiovascular disease. [7, 9–12] Exosomes provide an essential diagnostic tool because they can be detected early in cancer cells through non-invasive liquid biopsies [13] and may provide new therapeutic options for treatment-resistant, highly advanced, and metastatic cancers. [10, 14–18] There is no consensus on the biological mechanism of exosome biogenesis and cancer cell uptake; however, exosomes have been studied as routes to deliver drugs or substances to target cells. [3, 19]

RAB, a small GTPase, functions as intracellular and extracellular vesicle transfer machinery. [20–23] In particular, RAB27A and RAB27B are secretory RABs with each effector protein, increase intracellular transfer and plasma membrane docking of multivesicular bodies (MVBs) to secrete ILVs and exosomes. [24–26] miRNA, part of the exosome cargo, can be secreted extracellularly by RAB27A and RAB27B. [4, 5, 27] To date, 121 common miRNAs have been identified in exosomes. Theoretically, these miRNAs can interact with 24,000 mRNAs, each of which can interact with up to 200 mRNAs. Releasing only a few miRNAs that exert this regulatory effect may alter the biological pathway whereby cancer cells acquire metastatic potential. [3, 27, 28] Ostenfeld et al. suggested that, in the bladder cancer cells, RAB27A or RAB27B inhibited the secretion of miR23b and miR921, thereby reducing cancer cell invasion. Compared to the primary tumors in the metastatic lymph nodes, they found a reduction in miR-23b and miR-921 during metastasis. [26] Their experimental analysis revealed that two miRNAs were secreted via the action of RAB27A or RAB27B related to exosome secretion, potentially altering bladder cancer cell the metastatic potential [26]. Therefore, in this study, we examined the extent to which specific miRNAs are secreted as cargo within exosomes and how they affect RCC metastasis.

miRNAs act as tumor suppressor genes and selectively enter exosomes via cargo sorting. Cargo sorting occurs in various ways, depending on the substances in the exosomes. Exosome sorting of RNA cargo has been classified into three main categories: EXOmotif, Annexin-2, and Ago2, an RNA-induced silencing complex (RISC) protein. [29, 30] EXOmotifs are short-sequence motifs of exosomal miRNA that bind to hnRNPA2B1 to allow miRNAs to enter the exosome. [30] Hypothetically, the more specific miRNAs each EXOmotif has, the more likely miRNA sorting will occur in the exosome, and the more likely this will affect cancer cell metastasis. In other words, these miRNAs may have a greater chance of being secreted with the exosomes when RAB27A is overexpressed.

Previously, we found that negative RAB27A expression positively correlated with lymph node metastasis and poor prognosis in patients with RCC, as evaluated using tissue microarray samples; RAB27A may affect the metastatic potential of cancer cells [31]. In this study, we focused on the effects of exosome secretion and disposal of tumor suppressor genes on metastasis in RCC cells using in vitro cell experiments and bioinformatics studies. To our knowledge, this is the first study to use RAB27A overexpressing cells in RCC using novel methods, including ExoView analysis. This study aimed to investigate whether the exosome-mediated secretion of specific miRNAs can accelerate metastasis in RCC.

## Methods and materials

### Cell lines and reagents

Human renal cell carcinoma cell lines A498, ACHN, Caki-1, Caki-2, SN12C, SNU267, SNU349, and SNU482 were provided by Professor Jiyun Yoo (Gyeongsang National University, Jinju, Korea). The A498, ACHN, Caki-1, Caki-2, and SN12C cell lines were cultured in Dulbecco’s modified Eagle’s medium (DMEM, Gibco, #11,995–065, NY, USA), and the SNU267, SNU349, and SNU482 cell lines were cultured in RPMI 1640 (Gibco, #11,875–093). Both media were supplemented with 10% fetal bovine serum (FBS; Gibco, #26,140–079) and 1% penicillin–streptomycin (Corning, #30–002-CI, NY, USA), and the cell lines were incubated at 37 °C in an atmosphere containing 5% CO_2_.

### Semi-quantitative PCR

RNA was extracted from RCC cell lines using TRIzol reagent (Qiagen, Hilden, Germany). The total RNA was quantified using a NanoDrop 2000 spectrophotometer (Thermo Fisher Scientific, Waltham, MA, USA). The prepared RNA (1 μg) was reverse-transcribed to cDNA using the Maxime RT PreMix Kit (iNtRON, Burlington, MA, USA). Equal amounts of synthesized cDNA (1 μg) were used for semi-quantitative PCR using the Maxime PCR PreMix kit (iNtRON, #25,025). Primers for RAB27A (Bioneer, #P196767, Oakland, CA, USA), RAB27B (Bioneer, #P119176), and MYCN (Bioneer, #P282059) were used. PCR was performed in a 20-μL volume using a thermocycler (Biometra, Uberlingen, Germany) with the following PCR program: predenaturation for 2 min at 94 °C, denaturation for 20 s at 94 °C, annealing for 10 s at 58 °C, extension for 20 s at 72 °C, and a final elongation for 2 min at 72 °C. The PCR was performed for 40 cycles. PCR products were analyzed by electrophoresis on a 1.5% agarose gel, and band intensity was measured directly using a Gel Documentation System (Bio-Rad, Hercules, CA, USA).

### Western blotting

Proteins were extracted from harvested cells using RIPA lysis buffer (Thermo Fisher Scientific, #89,900) containing a protease inhibitor cocktail (Thermo Fisher Scientific, #78,430). The total protein concentration of each cell lysate was measured using the Bradford method, with bovine serum albumin as a standard. Equal amounts of protein lysate (45 µg) were loaded onto denaturing polyacrylamide gels and transferred to a nitrocellulose membrane. The primary antibodies used for immunoblotting were anti-RAB27A (diluted 1:2000; #ab55667; Abcam), anti-RAB27B (diluted 1:2000; Cat # PA5-54,096; Thermo Fisher Scientific), and anti-MYCN (diluted 1:500; Cat # ab24193; Abcam) followed by incubation with horseradish peroxidase-conjugated secondary antibodies. The immunoreactive bands were detected using enhanced chemiluminescence (Cat #32,109; Thermo Fisher Scientific). Digital chemiluminescent images were captured and analyzed using Fusion Solo (Vilber, Marne-la-Vallee Cedex, France).

### Knockdown of RAB27A and RAB27B

SNU482 cells were cultured to 70–80% confluence in 60-mm dishes and transfected using Lipofectamine 3000 (Invitrogen, #L3000015, Thermo Fisher) with human RAB27A siRNA (Bioneer, #5873–2) and negative control scrambled siRNA (Bioneer, #SN-1002) at a final concentration of 50 nM. The siRNA sequences were as follows: RAB27A,5’-CACAACAGUGGGCAUUGAU-3ʹ (sense), and 5ʹ-AUCAAUGCCCACUGUUGUG-3ʹ (antisense). After 24 h of incubation, the cells were retransfected using the above protocol. The cells were incubated for 72 h before harvesting. The experiments were independently repeated three times.

### Generation of RAB27A-overexpressing stable cell lines

ACHN cells were transfected with pCMV3-RAB27A plasmid (Cat # HG15862-UT; Sino Biological Inc.). Forty-eight hours after transfection and every 2–3 d after that, the cells were cultured with DMEM containing hygromycin B (Sigma) at a final concentration of 250 µg/ml. Parental ACHN cells (Korean Cell Line Bank, #21,611) were used as transfection controls.

### Cell migration assays

RAB27A knockdown SNU-482 cells were cultured in 24-well plates. For siRNA transfection, the cells were transfected twice using Lipofectamine 3000 transfection reagent as described above. When the cells reached 100% confluency, they were seeded in Culture-Insert 2 Well in µ-Dish 35 mm (Ibidi, GmBH, Grafelfing, Germany) and allowed to attach. Then, the cells were incubated at 37 °C under an atmosphere containing 5% CO_2_, and the divided area was monitored. JuLI™ Br (NanoEnTek, Waltham, Ma, USA) was used to capture images and monitor the migration of the cells, and images were analyzed by ImageJ. The experiments were independently repeated three times. The efficiency and maintenance of transfection were confirmed by western blot analysis. This process was repeated in RAB27A-overexpressing ACHN cells.

### Cell adhesion assay

RAB27A knockdown SNU-482 cells were seeded in 24-well plates and transfected twice with RAB27A siRNA. Ninety-six-well tissue culture plates were coated with basement membrane matrix (Matrigel, 354,234, BD Bioscience Franklin Lakes, NJ, USA) (16 μl per well, diluted 1:3 with serum-free media) for one h at 37 °C. The cells were blocked with 2% bovine serum albumin (2 μl per well) for two hours at 37 °C and washed twice. After detachment with trypsin, 12 wells of transfected RCC and parental RCC cells were plated on Matrigel-pre-coated 96-well plates in a serum-free medium. After 45 min, the wells were washed twice with PBS to remove the non-adherent cells. The cells in each well were stained with methylene blue and counted manually. The experiments were independently repeated three times. The efficiency and maintenance of transfection were confirmed by western blot analysis. This process was repeated in RAB27A-overexpressing ACHN cells.

### Transmission electron microscopy

Transmission electron microscopy (TEM) was used to characterize the histomorphological features of MVBs, which are the precursors of exosomes, in RAB27A-overexpressing cells. RAB27A-overexpressing and control cells were rinsed in 1 ml of PBS and fixed with 2.5% glutaraldehyde for 24 h at 4 °C. Cells were rotated for 30 min with 2% osmium tetroxide in PBS (1:1), dehydrated using a downgraded ethanol series for 30 min each, and embedded in Epon. Ultrathin sections were prepared and stained with uranyl acetate and lead citrate. Images were captured using a Bio 120 kV Transmission electron microscope (Talos L120C, Thermo Fisher Scientific,).

### RNA extraction and next-generation sequencing analysis

The RNA from RAB27A-overexpressing and control cells was pooled and extracted for RNA sequencing. Library preparation and validation quality checks were performed before next-generation sequencing. Ribodepletion was performed during library preparation using Ribo-zero H/M/R Gold. Using an Agilent Technologies 2100 Bioanalyzer, total RNA integrity was checked using an RNA integrity number value equal to or greater than 7. Template size distribution was checked on an Agilent Technologies 2100 Bioanalyzer using a DNA 1000 chip to verify the size of the PCR-enriched fragments. Transcriptome sequencing was performed using an Illumina platform. Raw RNA sequencing data were extracted as fragments per kilobase of exons per million fragments mapped (FPKM) for each sample.

### Protein–protein interaction and competing endogenous RNA (ceRNA) network analysis

Screening of RNA sequencing data from RAB27A-overexpressing cells identified 28 miRNAs with a fold-change of 17 and log(CPM) > 2. Genes associated with these 28 miRNAs were identified using TargetScan (http://www.mirdb.org/) and miRDB (http://www.mirdb.org/). The top 20 highest -ranking ceRNA networks were selected based on their scores presented on the portal. A ceRNA network was constructed using Cytoscape v. 3.9.1 (Cytoscape, RRID: SCR_003032) [32]. In addition, PPIs were analyzed in 167 protein-coding RNAs using STRING (https://string-db.org/): 167 selected RNAs had fold-change > 10, p <  − 0.05, and log(CPM) ≥ 2. The PPI maps show interactions with edge reliability ≥ 0.4. K-means clustering was used to divide the proteins into three groups (coded red, green, and blue in the map). For Gene Ontology enrichment analysis of the proteins identified via PPI, we analyzed the protein-coding RNAs used in the PPI analysis using ClueGO v. 2.5.9 (Cytoscape, San Diego, CA, USA). Only pathways with p < 0.05 are displayed.

### ExoView analysis

The biological and physical properties of exosomes in the ACHN cell line were characterized using ExoView R-100 (NanoView Bioscience, Boston, MA, USA) and ExoView Tetraspanin kits (EV-TETRA-C, NanoView Biosciences,), including anti-CD81, anti-CD63, anti-CD9, IgG negative control immobilized chips (NanoView Biosciences, EV-TETRA-C), fluorescent labeling agents, washing solutions (solution A and B) and blocking solution (NanoView Biosciences). The ExoView Tetraspanin Cargo kit (EV-TETRA-C, Nanoview Bioscience), including anti-syntenin antibody (Nanoview Bioscience, EV-TETRA-C-CA), cargo staining blocking solution, solution C, and solution D. Briefly, 35 µl of a diluted sample with solution A was dropped on the ExoView tetraspanin chip and incubated overnight (16 h) at room temperature. After incubation, the sample-loaded chip was washed twice with 1 ml of solution A, 250 mL of solution C, and 250 mL of solution D, three times for 3 min each. After the last wash, the exosomes on the chip were labeled by using 250 µl of a fluorescently labeled antibody mixture of anti-syntenin/AF 555 (green), anti-CD 63/AF 647 (red), and anti-CD 9/AF 488 (blue) (Nanoview Bioscience, EV-TETRA-C) and incubated for 1 h at room temperature. The co-localization of tetraspanin on the surface and the cargo of exosomes was measured. During this process, anti-CD 63/AF 647 and anti-CD 9/AF 488 were diluted 1:500, and syntenin/AF 555 (Nanoview Bioscience, EV-TETRA-C-CA) was diluted 1:200 in a mixture of solution A and a cargo-blocking solution. Finally, the chip was rinsed with 1 ml of solutions A and B 3 times for 3 min each and dried at room temperature. The exosome-captured chip was scanned by Exoview R-100 using nScan software, and the data were analyzed using Exoview Analyzer 3.0 software. We repeated the process using a mature miR beacon, rather than syntenin, as the cargo. We designed the molecular beacons to be complementary to the mature nucleotide sequence of miR-127-3p, according to the protocols of Baker et al. [33] and Obernosterer et al.[34], with minor modifications. The beacons contained 22 complementary bases (UCGGAUCCGUCUGAGCUUGGCU), a 6FAM fluorophore on the 5’-end, and a black Hole Quencher (BHQ1) on the 3’-end.

### Cell uptake experiment

ACHN cells (parental cells) and RAB27A-overexpressing ACHN cells were cultured in an exosome-free medium (exosome-depleted FBS; #A2720803, Thermo Fisher Scientific) supplemented with 10% fetal bovine serum (#26,140–079; Gibco; Thermo Fisher Scientific) and 1% penicillin–streptomycin (Cat #30–002-CI, Corning, NY, USA). Cell lines were incubated for 48 h at 37 °C under an atmosphere containing 5% CO_2_. Media from parental and RAB27A-overexpressing ACHN cells were harvested, added to each parental cell line, and incubated for 48 h. When the cells reached 100% confluency, they were seeded in Culture-Insert 2 Well in µ-Dish 35 mm (Ibidi, GmBH, Grafelfing, Germany) and allowed to attach. Then, the cells were incubated at 37 °C under an atmosphere containing 5% CO_2_, and the divided area was monitored and analyzed using JuLI Br (NanoEnTek, Waltham, Ma, USA). We repeated this process using RAB27A-overexpressing cells as recipient cells.

### Statistical analysis

Data are presented as mean ± standard deviation. Differences between control and experimental groups were analyzed using a two-tailed Student’s t-test. In addition, the statistical significance of the fold change in the transcript expression profile in the bioinformatics studies was determined using paired t-tests. Statistical significance was set at p < 0.05.

## Results

### RAB27A and RAB27B mRNA and protein expression in renal cell carcinoma

**Fig. 1:**
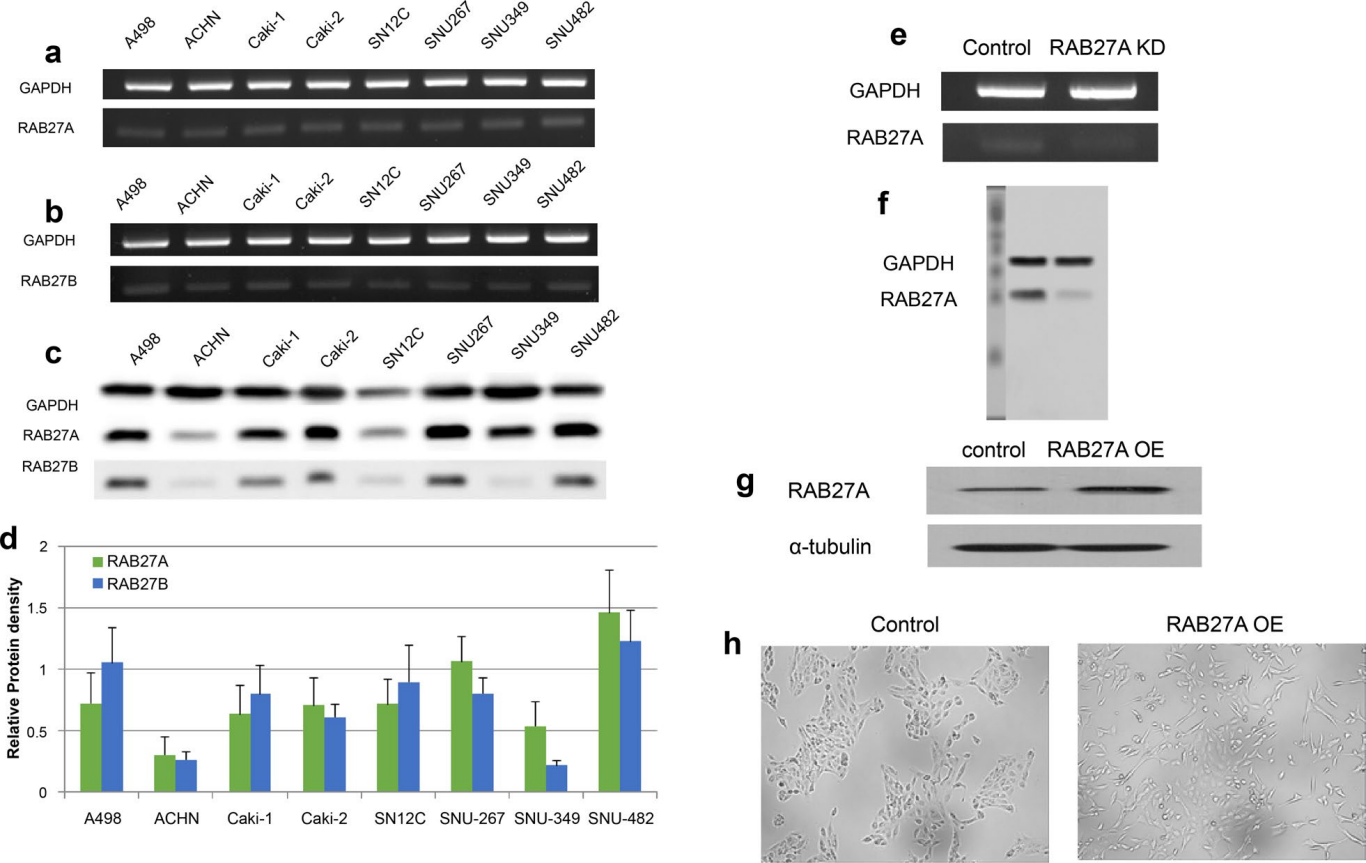
Identification of RAB27A and RAB27B in renal cell carcinoma (RCC). **a** RAB27A mRNA expression was evaluated in eight RCC cell lines, including A498, ACHN, Caki-1, Caki-2, SN12C, SNU267, SNU349, and SNU482. **b** RAB27B mRNA expression was evaluated in eight RCC cell lines. **c**, **d** A high level of RAB27A protein expression was observed in A498, Caki-1, Caki-2, SNU267, SNU349, and SNU482 cells. **e**–**f** After treatment with siRNA, decreased levels of RAB27A mRNA and protein were observed in the SNU482 cell line. **g** Increased levels of RAB27A protein were observed in the ACHN and RAB27A-overexpressing cells by western blotting. **h** RAB27A-overexpressing renal cell carcinoma cells had a different morphology than the ACHN parental cells; these cells had an elongated fibroblast-like spindle-shaped morphology with an increased abundance of cytoplasm

The mRNA expression levels of RAB27A and RAB27B mRNA were evaluated in eight renal cell carcinoma (RCC) cell lines. As shown in Fig. 1a and 1b, RAB27A mRNA was expressed at relatively higher levels than RAB27B mRNA. Western blotting confirmed that RAB27A and RAB27B proteins were expressed. (Fig. 1c and d). Because mRNA and protein expression of RAB27A were high in SNU482 cells, we used them for the RAB27A knockdown (KD) study. Decreased levels of mRNA (Fig. 1e) and protein (Fig. 1f) expression of RAB27A were observed in SNU482 cells after treatment with siRNA sequences. Since mRNA and protein expression of RAB27A were the lowest in ACHN cells, we used these cells for RAB27A overexpression (OE) studies. After generating a RAB27A OE stable cell line (ACHN), we confirmed protein expression by western blotting (Fig. 1g). RAB27A OE cells had a morphology different from control cells, exhibiting an elongated fibroblast-like spindle-shaped morphology with more abundant cytoplasm (Fig. 1h).

### RAB27A silencing showed a nonspecific change in cell migration but an increase in adhesion

**Fig. 2:**
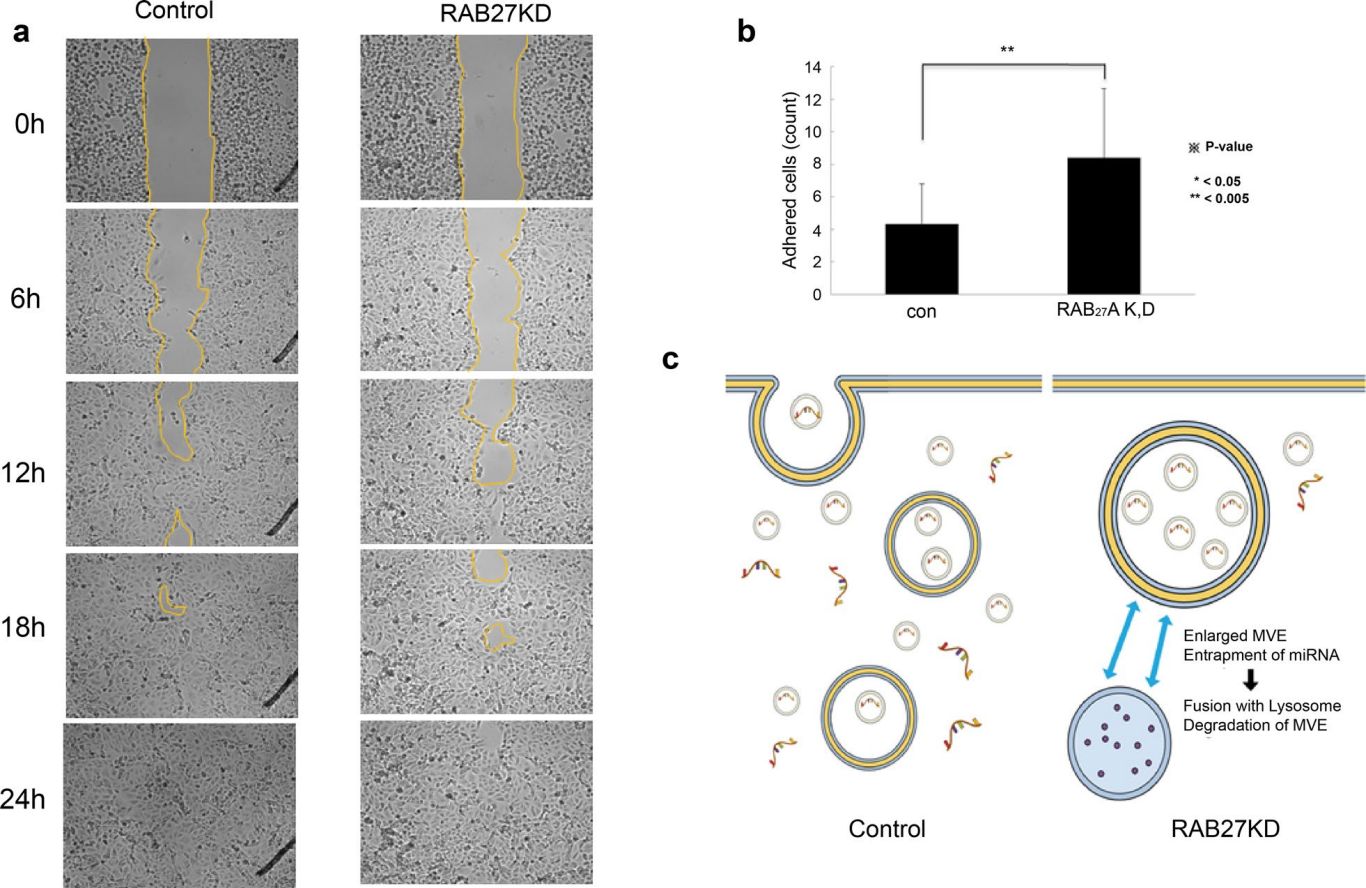
**a **RAB27A siRNA treatment of SNU482 cells induced a nonspecific change in migratory activity compared to that in the control cells. **b** The average number of adhered cells was 8/high power field (HPF) in the RAB27A KD group but 4/HPF in the control group (with specific targeting of RAB27A). **c** When multivesicular endosomes are enlarged, they fuse with lysosomes and are degraded with internal genetic materials, especially tumor suppressor genes

The gap closure caused by the migration of SNU482 knockdown (KD) and control cells over time was quantified at 0, 6, 12, 18, and 24 h. The gap between the two groups was closed at 18 h, suggesting that RAB27A KD induced a nonspecific change in migratory activity. (Fig. 2a). When the number of adhered cells was quantified, RAB27A KD in SNU482 cells increased in cell adhesion compared to that in the control group. The average number of adhered cells was 8/high power field (HPF) in the RAB27A KD group and 4/HPF per in the control group (Fig. 2b). In this study, the mild increase in adhesion in the RAB27A KD cells was probably due to the function of other secretory RABs, such as RAB8 or RAB11, on exosomal release or the enlargement of MVBs to fuse with lysosomes to be degraded with their tumor suppressor genes, miRNAs, to obtain metastatic potential scarcely (Fig. 2c).

### RAB27A overexpression accelerated cell migration and adhesion

**Fig. 3:**
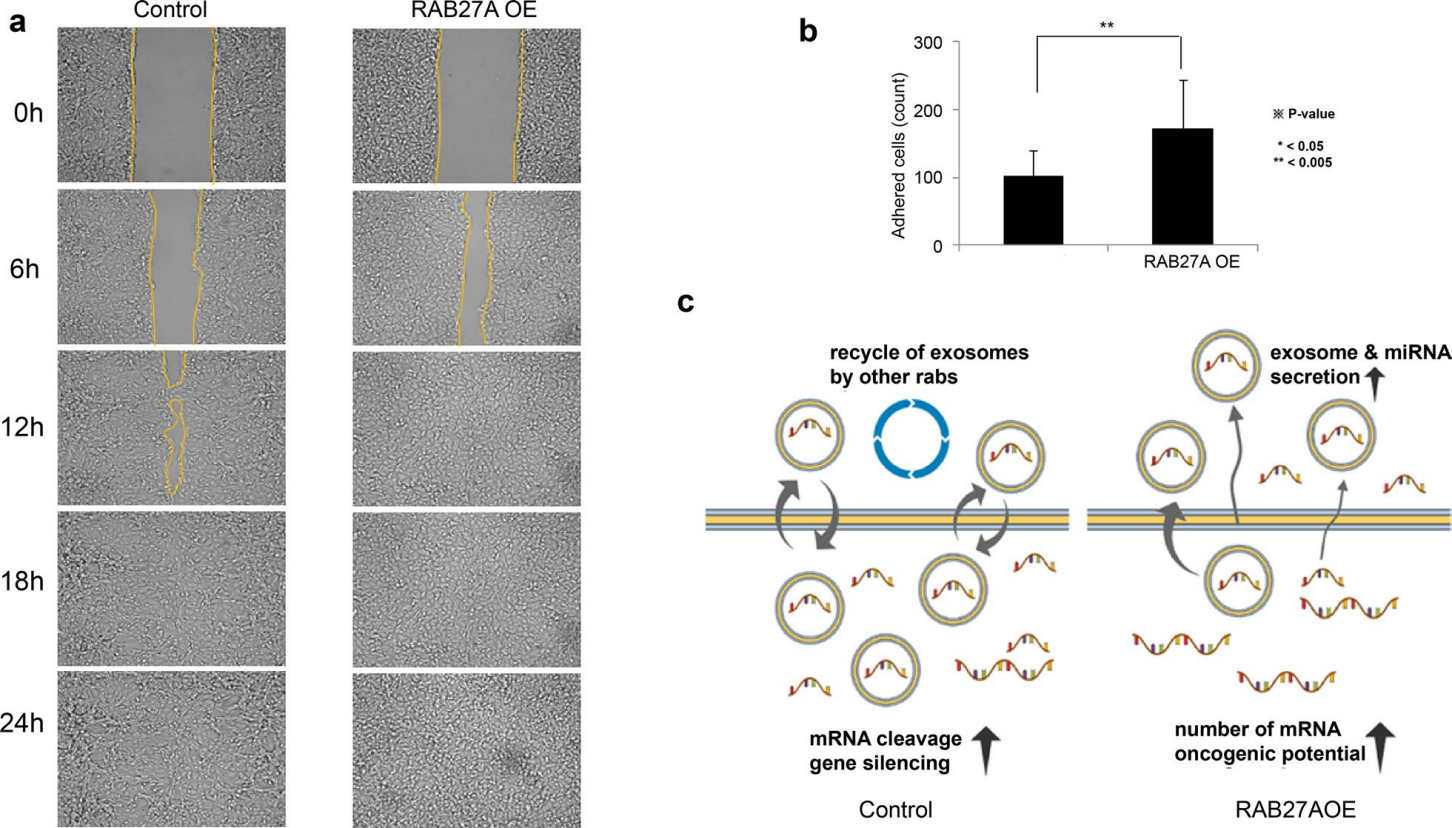
**a **During the 24-h cell migration assay, there were significant differences between the RAB27A-overexpressing group and the control group. **b** The average number of adhered cells was 190/HPF in the RAB27A KD group and 100/HPF in the control group. **c** RAB27A overexpression accelerates exosome secretion to dispose of miRNAs, increasing the metastatic potential of RCC cells by enhancing the induction of target genes. In contrast, the target genes are cleaved and silenced in the control cells when the balance and recycling of exosomes and other RABs are maintained

During the 24-h cell migration test, there were significant differences between the RAB27A OE and control groups, and the gap was closed at 12 h in the RAB27A OE group. (Fig. 3a). A significant difference was observed in the cell adhesion test, in which the average number of adhered cells was 190/high power field (HPF) in the RAB27A OE group and 100/HPF in the control group (Fig. 3b). The increase in migration and adhesion in the RAB27A OE group was probably due to the function of RAB27A in increasing exosome release, leading to the extraction of tumor suppressor genes and miRNAs extracellularly compared to the control group with recycling of exosomes containing cargo genes (Fig. 3c).

### The size distribution of exosomes of RAB27A OE and the control group showed no significant difference

The size distribution of the exosomes from the RAB27A OE and control groups ranged from 50 to 200 nm. The mean size distribution of exosomes in the RAB27A OE group captured by the tetraspanin capture probe was 58.67 ± 14 nm for CD63, 63.67 ± 21.67 nm for CD81, 61 ± 18 nm for CD9, and 70 ± 28.67 nm for MIgG. The size distribution of exomes in the control group captured by the tetraspanin capture probe was 60 ± 18 nm for CD63, 67 ± 26 nm for CD81, 60 ± 18 nm for CD9, and 66 ± 27 nm for MIgG. Data are presented as mean ± standard deviation. There was no significant difference in the exosome size between the RAB27A OE and control groups.

### The number of exosome particles in RAB27A overexpressing cells tended to be higher than those in the control group

**Fig. 4:**
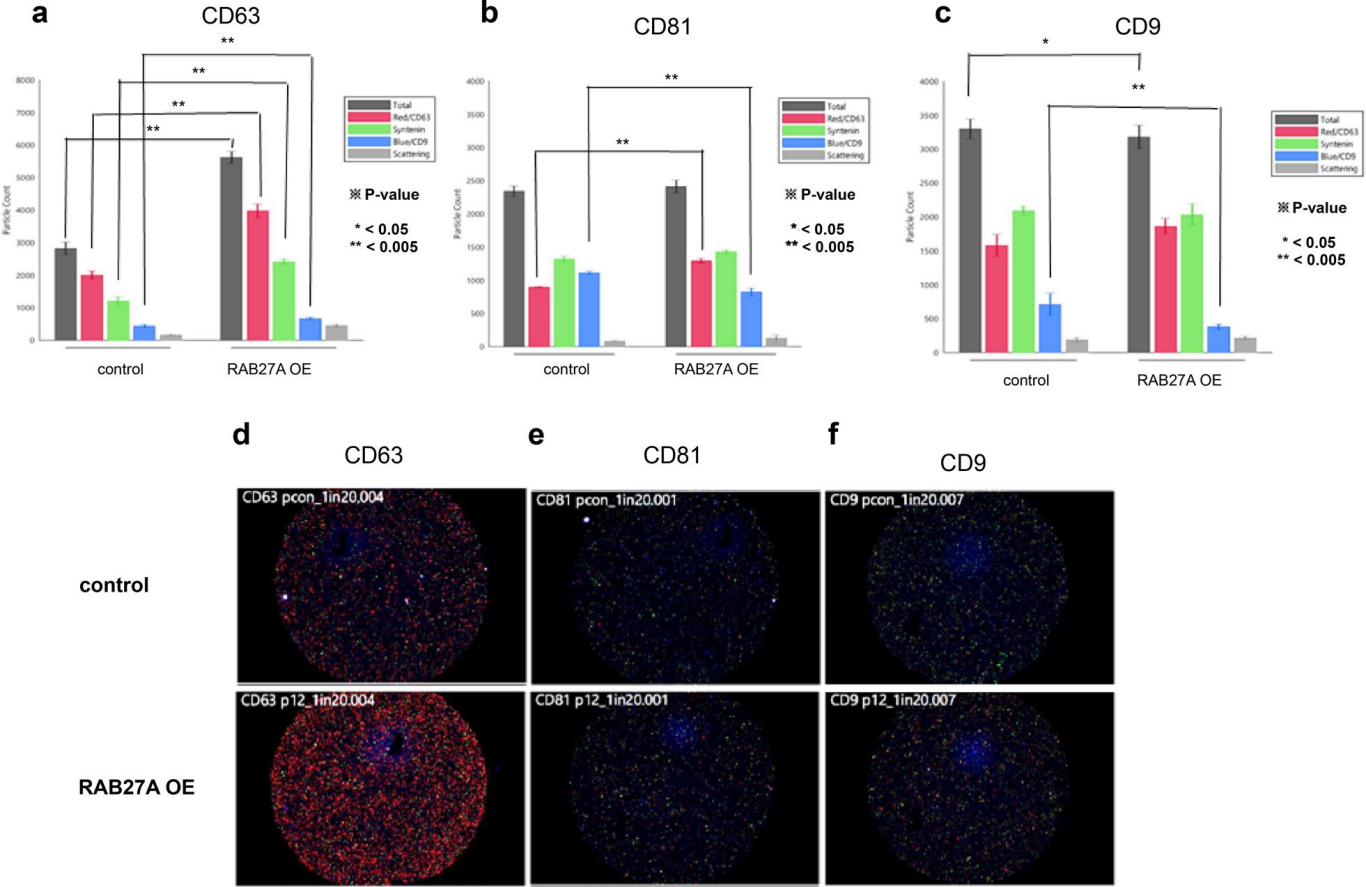
Particle counts. The particle counts of exosomes from RAB27A-overexpressing ACHN and control cells were determined and summed according to each tetraspanin capture spot, namely, **a** CD63, **b** CD81, and **c** CD9, and to each capture antibody, namely, CD63 (red bar), Syntenin (green bar), and CD9 (blue bar). The exosome particle count in RAB27A-overexpressing ACHN cells was significantly higher than that in the corresponding control cells. The representative images of each particle count of exosomes from RAB27A-overexpressing ACHN cells and the corresponding control cells are visualized according to each tetraspanin capture spot, namely, **d** CD63, **e** CD81, and **f** CD9

The particle counts of exosomes of the RAB27A OE and control groups were determined and numbered according to each tetraspanin capture spot, including CD63, CD81, and CD9, and each capture antibody, including CD63, syntenin, and CD9, as shown in Figs. 4a-c, respectively. The number of exosomal particles in the RAB27A OE group was higher than in the control group. Images of representative capture spots, including CD63, CD81, and CD9, for the control and three different passages of the RAB27A OE group are shown in Fig. 4d–f. Among the tetraspanin capture spots, CD63 showed the highest number of exosomes, followed by CD81 and CD9. For grouped and pooled data, the average total number of particle counts of exosomes collected from RAB27A OE was 5395 ± 465.2239 for CD63, 2359.333 ± 138.4007 for CD81, and 3030.889 ± 207.769 for CD9, which were significantly higher than those of the control group, 2820 ± 198.4616 for CD63, 2337.333 ± 78.7676 for CD81, and 3296 ± 146.5026 for CD9. The particle counts for each captured antibody are listed in Table 1.

**Table 1:** Particle counts of exosomes of RAB27A overexpressing cells

Capture spot	Capture antibody	Control	RAB27A OE	P-value
CD63	Total	2820 ± 198.4616	5395 ± 465.2239	**1.91403E-06**
	CD 63	2001 ± 128.1601	3901.333 ± 301.7308	**5.88789E-07**
	Syntenin	1193.333 ± 137.0596	2118.444 ± 256.025	**8.03985E-05**
	CD 9	432 ± 56.15158	609.3333 ± 64.29813	**0.00085983**
	Scattering	155.3333 ± 31.56475	407.8889 ± 88.40312	**0.00041037**
CD81	Total	2337.333 ± 78.76759	2359.333 ± 138.4007	0.40141881
	CD 63	895 ± 11.53256	1164 ± 113.9561	**0.001356952**
	Syntenin	1313.667 ± 46.00362	1336 ± 134.5678	0.394699851
	CD 9	1112.333 ± 22.05297	837.5556 ± 63.30899	**1.51438E-05**
	Scattering	80.66667 ± 15.17674	103.6667 ± 36.59235	0.163160366
CD9	Total	3296 ± 146.5026	3030.889 ± 207.7694	**0.035600379**
	CD 63	1577.667 ± 161.9887	1682.444 ± 169.5458	0.185865817
	Syntenin	2092.333 ± 68.98067	1897.444 ± 197.5045	0.067059633
	CD 9	707.3333 ± 161.7168	402.8889 ± 45.37192	**0.000129677**
	Scattering	183 ± 39.15354	181.5556 ± 32.84095	0.475364719

### RAB27A overexpression increases the number of multivesicular bodies and intraluminal vesicles

**Fig. 5:**
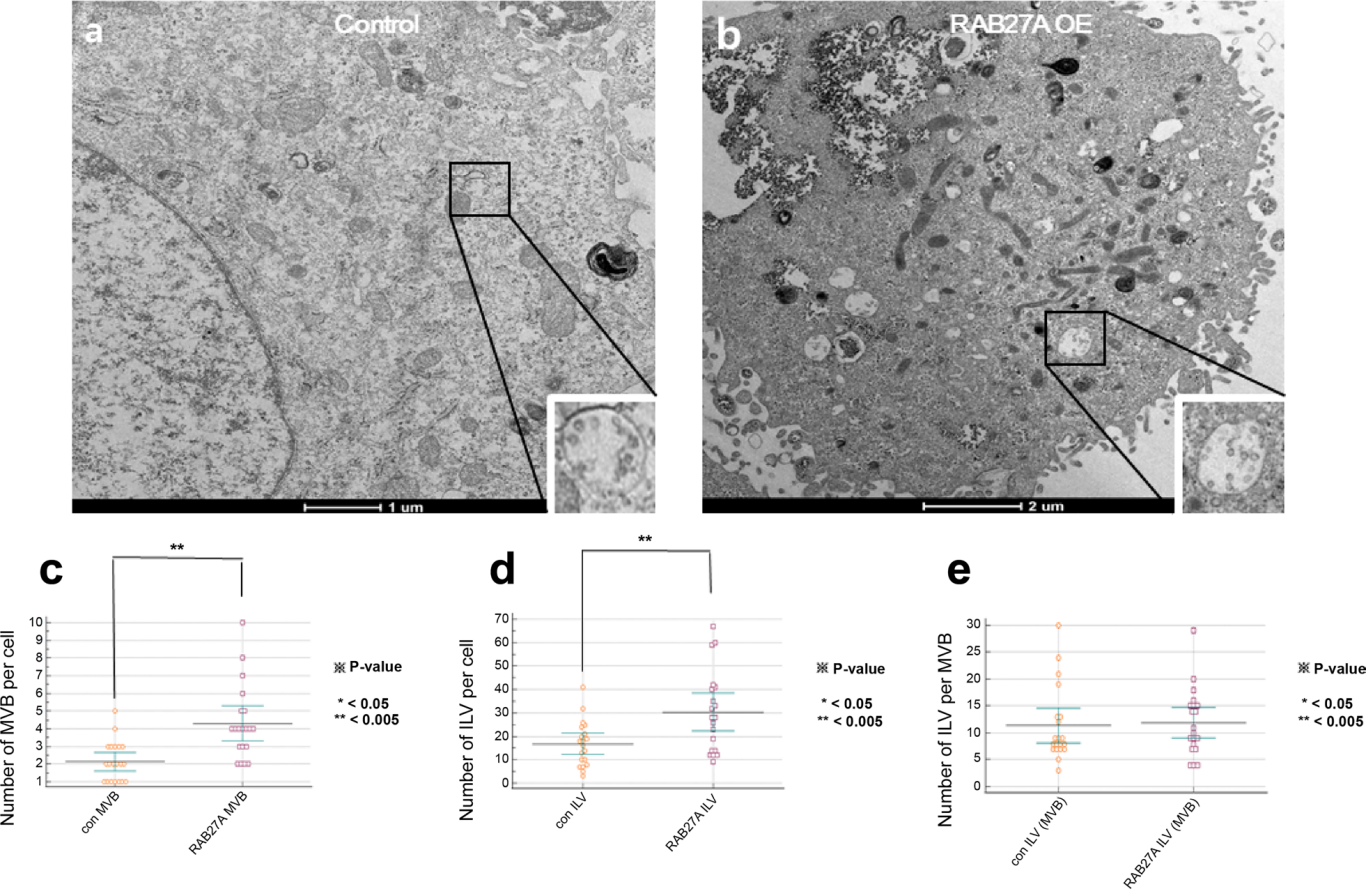
Morphology and size of Multivesicular bodies (MVBs) and intraluminal vesicles (ILVs). **a**, **b** The morphology and size of MVBs and ILVs in RAB27A-overexpressing cells **b** showed no significant differences compared to those in control cells **a**. In contrast, **c** the number of MVBs and **d** ILVs per cancer cell were significantly increased in the RAB27A overexpressing cells compared to the control cells. **e** The number of ILVs per MVB showed no significant increase

To investigate the mechanism of increased exosome secretion in RAB27A OE RCC cells, electron microscopy was performed to evaluate the characteristics of ILV and MVEs. Although exosome secretion was dramatically increased in RAB27A OE cells as described above, the morphology and size of MVBs and ILVs in RAB27A OE cells (Fig. 5a) did not show significant changes compared to those in the control group (p = 0.3964) (Fig. 5b). However, the number of MVBs (Fig. 5c) and ILVs per cell (Fig. 5d) significantly increased in RAB27A OE cells (p = 0.0002 and p = 0.0024, respectively). The number of ILVs per MVB did not significantly increase (Fig. 5e).

### Identification of miRNAs and protein-coding genes in RAB27A-overexpressing cells

**Fig. 6:**
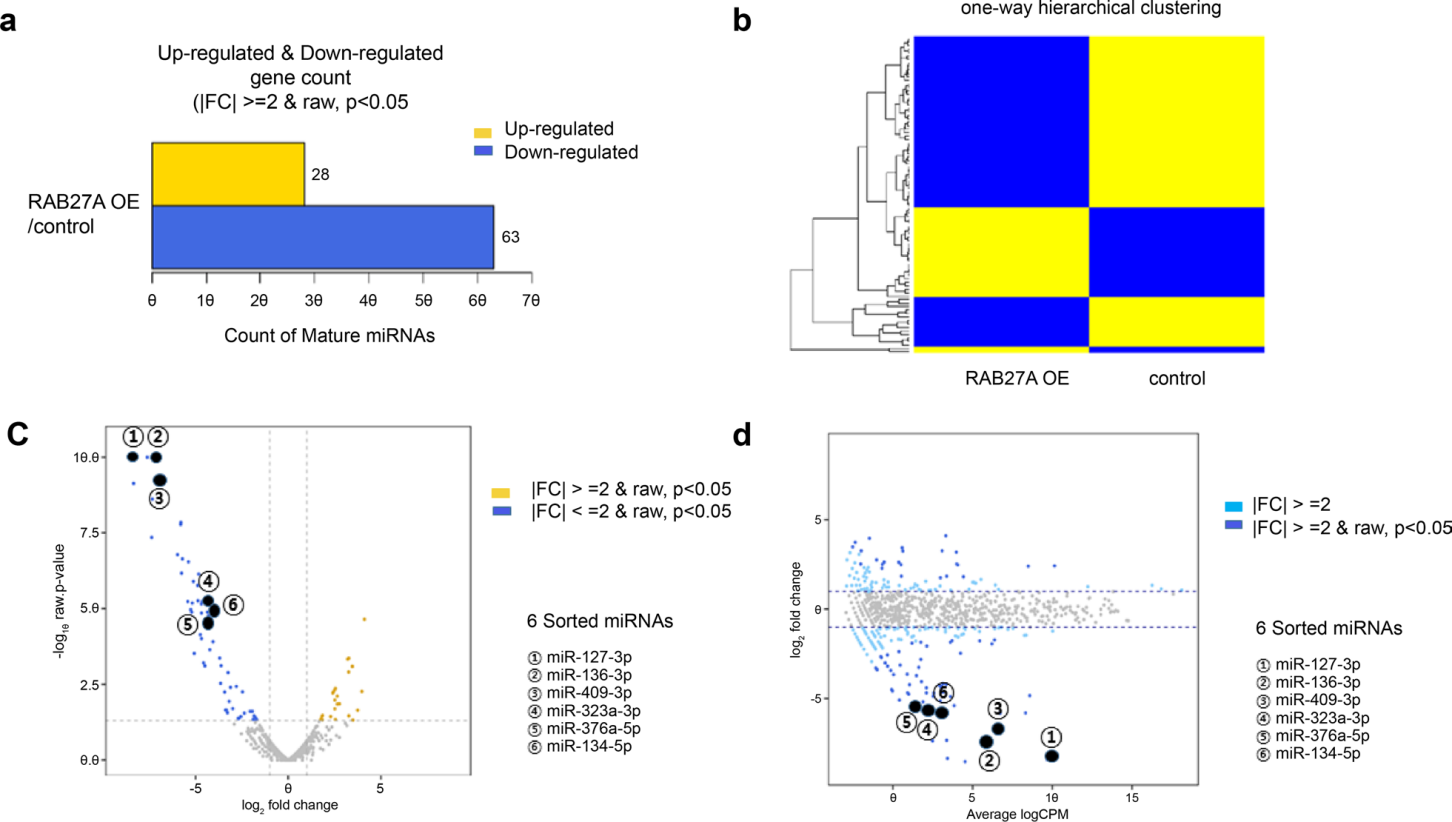
Changes in miRNA expression. **a** Twenty-eight upregulated and 63 downregulated miRNAs showed a significant difference in RAB27A overexpressing cells compared to control cells. (p < 0.05) **b** The heat map of the one-way hierarchical clustering results shows four clusters of differentially upregulated and downregulated genes between the RAB27A overexpressing and control cells. **c** The volcano plot shows upregulated miRNAs on the right (yellow) and downregulated miRNAs on the left side of the volcano plot (blue). **d** The smear plot shows 91 significantly differentially expressed (either upregulated or downregulated) miRNA genes in RAB27A-overexpressing cells compared to the corresponding ACHN control cells. There are six representative downregulated genes, including miR-127-3p, miR-136-3p, miR-409-3p, miR-376a-5p, and miR-134-5p, with sequences of Exomotif, and miR-323a-3p, related to Ago2, indicated in the volcano and smear plots

Using RNA sequencing, we detected 21,551 genes associated with RAB27A OE. Among the 327 miRNAs, 28 upregulated and 63 downregulated miRNAs were detected in the RAB27A OE group, which showed a significant difference from the control group. (fold change > 2 or < -2, raw). p < 0.05) (Fig. 6a). In the one-way hierarchical clustering heat map using the Z-score for normalizing values, there were four clusters of upregulated and downregulated genes between the RAB27A OE and control groups (Fig. 6b). The volcano plot evaluated the log2-fold change and p-value obtained from the comparing the two groups (X-axis: log2-fold fold change, Y-axis: -log10 p-value). The upregulated miRNAs are shown in the right region of the volcano plot (yellow), whereas the downregulated miRNAs are shown in the left region (blue) (Fig. 6c). A smear plot was drawn based on the overall average expression level to confirm the genes that showed higher expression differences in the RAB27A OE group (X-axis: Average log CPM; Y-axis: log2 Fold Change). Ninety-one differentially expressed miRNA genes, either up-regulated or down-regulated, were detected according to fold-change > 2 or < -2, raw criteria. p < 0.05 in the RAB27A OE group (Fig. 6d). Additionally, 14,299 protein-coding genes were associated with the RAB27A OE group. Among them, 1253 protein-coding genes showed a greater than twofold change with a significant difference from the control group (p < 0.05).

### Identification of MYCN as the most significant hub and association of miR-127-3p with MAPK4 in RAB27A overexpressing cells

**Fig. 7:**
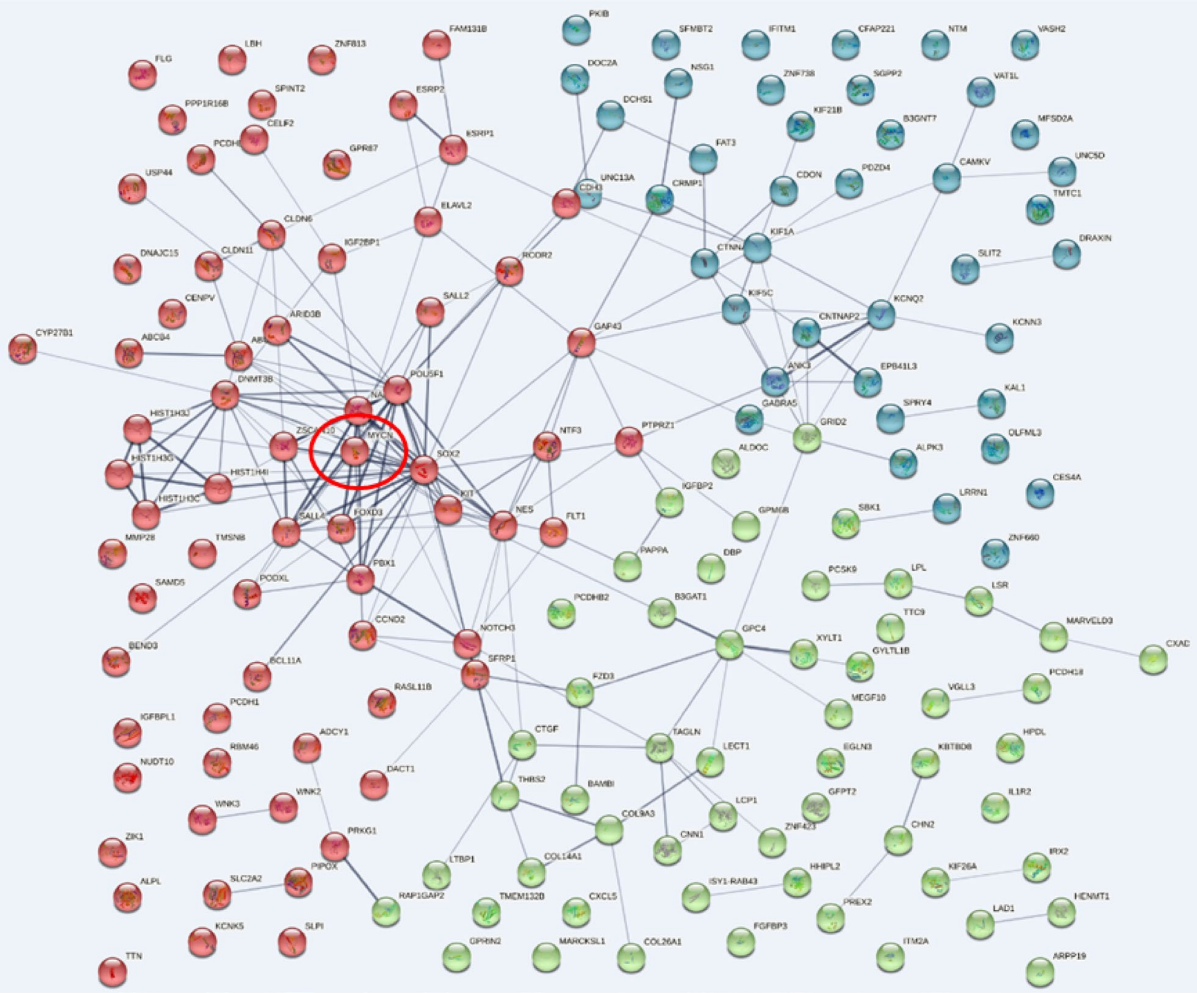
Protein–protein interaction. **a** We submitted 167 protein-coding genes (with RNA sequences exhibiting fold change > 10, p < 0.05, and a log(CPM) ≥ 2) to protein–protein interaction analysis in STRING. K-means cluster analysis divided the genes into three groups (red, green, and blue). MYCN (red circle) was identified as the most significant hub gene for renal cell carcinoma

**Fig. 8:**
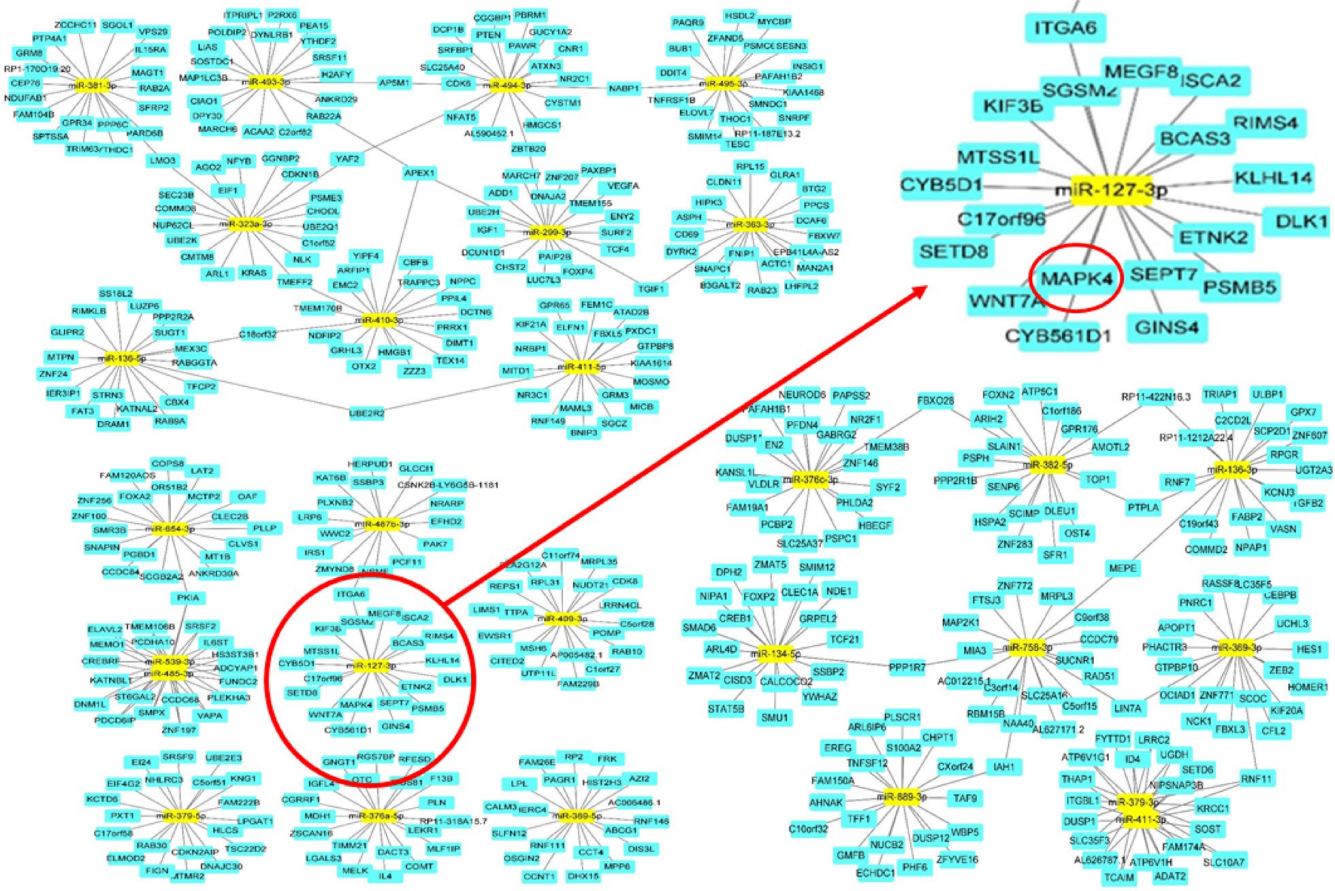
miRNA network analysis. A complete image of 28 miRNAs, including 2 clustered miRNAs, were used in the ceRNA network analysis. Of the 28 representative miRNAs in RAB27A overexpressing cells, miR-127-3p, one of the most representatively downregulated genes with a fold change of 339, showed a close relationship with MAPK4, indicated in a red circle

Using RNA sequencing data and protein–protein interactions (PPI), we analyzed 167 protein-coding RNAs of the RAB27A OE group with values of more than tenfold change, p < 0.05, and log CPM 2 or greater. The edge reliability was displayed from 0.4 or higher, and each data point was divided into three groups using K-means cluster analysis. Among these genes, MYCN was the most significant hub gene in RAB27A OE cells. (Fig. 7) PPI with gene ontology (GO) term analysis was conducted using ClueGO software. In addition, based on RNA sequencing data, 28 miRNAs with a 17-fold change and 2 < log CPM were identified in the RAB27A OE group. The genes associated with the 28 miRNAs were searched using TargetScan and miRDB, and ceRNA networks were produced based on the scores presented in Cytoscape 3.9.1. Figure 8 shows a full image of the 28 miRNAs, including two clustered miRNAs. Of the 28 miRNAs identified in RAB27A OE cells, miR-127-3p was associated with MAPK4. (marked in Fig. 8) We experimentally determined that MYCN protein expression was higher, but its mRNA expression lower, in RAB27A-OE cells than in the control ACHN cells (Additional file 1). Cell blocking of RAB27A-OE and control cells, with immune-staining for MYCN, revealed greater cytoplasmic expression of MYCN in the RAB27A-OE cells (Additional file 1). Given that miR-127-3p was one of the most representatively downregulated genes with a fold change 339, we further evaluated miR-127-3p with molecular beacon using Exoview.. By applying our previous results on exosomal RNA cargo sorting to this analysis of the RAB27-OE group, we then identified six candidate miRNAs (among the 28 representative identified) that probably undergo cargo sorting into exosomes. [28 29] Five genes, including miR-127-3p, miR-136-3p, miR-409-3p, miR-376a-5p, and miR-134-5p, had sequences of Exomotif [30], and miR-323a-3p was found to be related to Ago2 [29], as indicated in the volcano and smear plots in Fig. 6c and d.

### The number of exosome particles containing miR-127-3p in RAB27A overexpressing cancer cells was higher than in the control group.

**Fig. 9:**
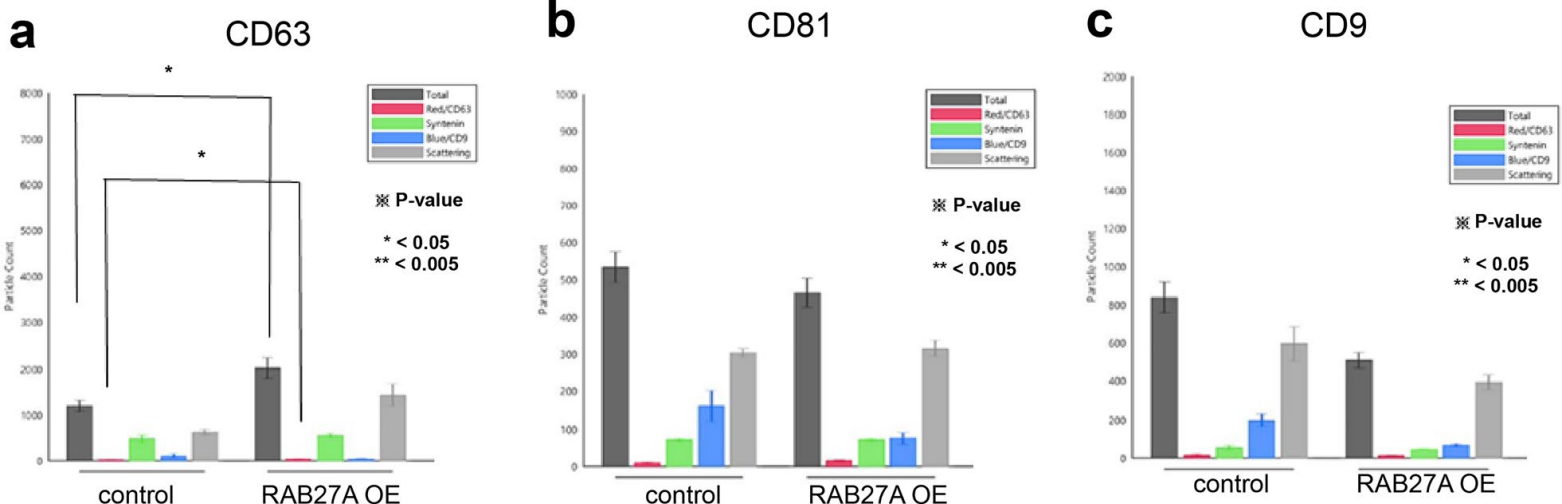
Analysis of miRNA exosomal cargo. The representative downregulated mature miRNA, hsa-miR-127-3p, was used as a fluorescent RNA probe of secreted miRNA exosomal cargo for Exoview analysis. Representative capture spots, namely, **a** CD63, **b** CD81, and **c** CD9, for RAB27A-overexpressing ACHN cells compared to the corresponding control cells. The particle counts of exosomes in the capture spot CD63 collected from RAB27A-overexpressing ACHN cells were significantly higher than those collected from the corresponding control cells

The representative downregulated mature miRNA in RAB27A OE cells, miR-127-3p, was used as a fluorescent RNA probe for the ExoView analysis. The particle counts for representative capture spots, including CD63, CD81, and CD9, for hsa-miR-127-3p are shown in Fig. 9a–c, respectively. For grouped and pooled data for captured hsa-miR-127-3p using fluorescence RNA probe, the average total number of particle counts of exosomes collected from the RAB27A OE cells was 2510.111 ± 816.550 for CD63, 587.333 ± 112.035 for CD81, and 832.444 ± 360.473 for CD9 and those of control group were 1199.667 ± 124.456 for CD63, 534.333 ± 40.526 for CD81, and 838.667 ± 81.298 for CD9. Among these particle counts, the CD63 count of RAB27A OE cells was significantly higher than that of the control group. The particle counts for each captured antibody are listed in Table 2.

**Table 2:** Particle counts of exosomes that carry miRNA of RAB27A overexpressing cells

Capture Spot	Capture antibody	Control	RAB27A OE	P-Value
CD63	Total	1199.667 ± 124.4562	2510.111 ± 816.55	**0.011474**
	CD 63	14 ± 5.196152	31.77778 ± 11.90355	**0.017216**
	Syntenin	480 ± 84.64042	649.3333 ± 194.857	0.092422
	CD 9	106.6667 ± 43.65012	438 ± 745.9501	0.236828
	Scattering	627.6667 ± 58.04596	1481.889 ± 229.2708	**5.08E-05**
CD81	Total	534.3333 ± 40.52571	587.3333 ± 112.0346	0.226533
	CD 63	10.33333 ± 2.309401	13 ± 3.5	0.126425
	Syntenin	71.33333 ± 3.21455	66.66667 ± 11.30265	0.254295
	CD 9	160.6667 ± 40.37739	159.3333 ± 94.38617	0.490987
	Scattering	303.3333 ± 11.01514	362.5556 ± 54.34637	**0.049534**
CD9	Total	1707.556 ± 604.3676	832.4444 ± 360.4733	0.488809
	CD 63	590.1667 ± 245.9087	18.55556 ± 11.19276	0.323706
	Syntenin	744.8889 ± 279.5393	61.33333 ± 23.28626	0.303812
	CD 9	1047.611 ± 402.4272	254.8889 ± 264.7803	0.362542
	Scattering	71.11111 ± 24.07559	516.8889 ± 124.9004	0.175221

### Renal cell carcinoma cells incubated with exosome-containing media showed an increase in migration, which proves the exosome uptake of cancer cells

**Fig. 10:**
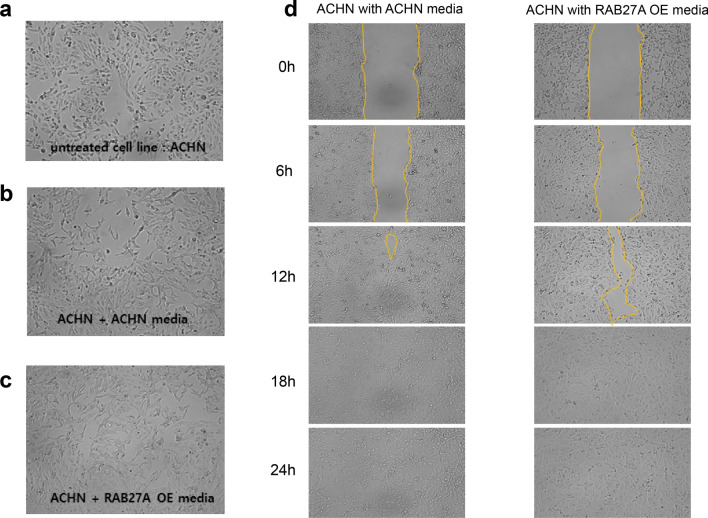
Cellular uptake analysis on ACHN cells. The cell uptake experiment used **a** parental ACHN cells incubated in two different media: medium from **b** parental ACHN cells and **c** medium from RAB27A-overexpressing ACHN cells. Morphological evidence showed the differentiation of ACHN cells into typical fibroblast-like cells with multipolar structures. **d** ACHN cells incubated with parental cell medium showed increased migration activities compared to cells incubated with the RAB27A-overexpressing cell medium

**Fig. 11:**
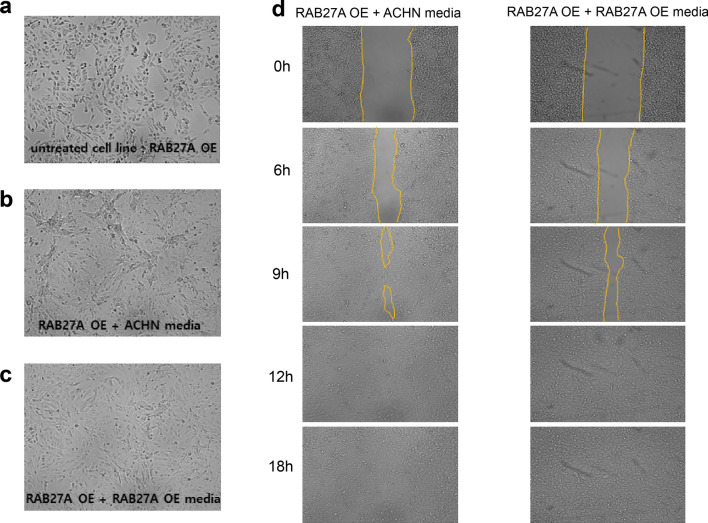
Cellular uptake analysis on RAB27A OE cells. Cell uptake experiment using **a** RAB27A OE cells incubated in two different media: medium from **b** parental ACHN cells and **c** medium from RAB27A-overexpressing ACHN cells. Morphological evidence showed that the fibroblast-like phenotype was more prominent in RAB27A-overexpressing cells incubated with **b** parental ACHN than in those incubated with **c** RAB27A-overexpressing cells. **d** RAB27A-overexpressing cells incubated with the parental ACHN cell medium showed increased migration activities compared to those incubated with the RAB27A-overexpressing cell medium

The migratory activity of RAB27A OE and control RCC cells was evaluated by incubating the cells in different exosome media. Following incubation in two different exosome media from control and RAB27A OE cells (Fig. 10a), the fibroblast-like phenotype was more prominent in control cells than in RAB27A OE cell exosome media. (Fig. 10b and c). Regarding migration activity, control cells with control cell-derived exosome media showed an increase in migration compared to RAB27A OE cell-derived exosome media (Fig. 10d). Next, following incubation in two different exosome media, those of control and RAB27A OE cells, in RAB27A OE cells (Fig. 11a), the fibroblast-like phenotype appeared more prominent in the control cell exosome media than in the RAB27A OE cell exosome media (Fig. 11b and c). Regarding cell migration activity, RAB27A OE cells treated with control cell-derived exosome media showed increased migration compared to RAB27A OE cell-derived exosome media. (Fig. 11d). Cells treated with control cell-derived exosome media showed increased migration compared to RAB27A OE cell-derived exosome media. Considering that the RAB27A OE cell exosome media contained more tumor suppressor miRNAs, the cellular uptake of these exosomes probably inhibited cancer cell metastasis.

## Discussion

This study used RAB27A knockdown (KD) and overexpression (OE) RCC cells to examine cell migration and adhesion. Ostrowski et al. found that exosomes secreted by RAB27A KD cells showed morphology and size identical to those of control cells, as assessed by electron microscopy. However, the size of the MVEs in RAB27A KD cells was significantly increased. [22] When endocytosed, most cell-surface receptors are packaged into small ILVs to generate MVBs, which are delivered to lysosomes for degradation. Less frequently, when RAB27A is not depleted, exosomes are generated by the direct fusion of MVBs with the plasma membrane. [23] In this study, the mild increase in adhesion in the RAB27A KD cells was probably due to the function of other secretory RABs, such as RAB8 or RAB11, on exosomal release [23] or the enlargement of MVBs to fuse with lysosomes to be degraded with their tumor suppressor genes, miRNAs, to obtain metastatic potential scarcely [22] (Fig. 2c). The increase in migration and adhesion in the RAB27A OE group was probably due to the function of RAB27A in increasing exosome release, leading to the extraction of tumor suppressor genes and miRNAs extracellularly compared to the control group with recycling of exosomes containing cargo genes [22, 23] (Fig. 3c).

Previously, Song et al. suggested that KIBRA inhibited the degradation of RAB27A and that KIBRA increased exosome secretion. In contrast to our study, they found that KIBRA depletion increased the MVB size and number. In KIBRA-knockdown cells, the number of MVBs per cell and ILVs per MVB increased, indicating an abnormal accumulation of ILVs in MVBs. [33] Together, in our study, since there was no size increase in MVBs or a significant increase in ILVs per MVBs, but an increase in the number of individual ILVs and MVBs, this indicates that RAB27A OE increases exosome secretion rather than degradation.

Until recently, cell experiments using RAB27A have been conducted in several other carcinomas. [12–14, 22, 24, 27] To the best of our knowledge, this is the first study to examine RCC cells overexpressing RAB27A. In cell experiments, RAB27A overexpressing cells showed increased metastatic potential in migration, adhesion assays, and cancer cell differentiation. RAB27A overexpressing cells had a more significant impact on metastasis than RAB27A KD in cell migration and adhesion, and RAB27A knockdown and overexpression in RCC cells did not have the opposite effect.

Bierings et al. suggested that the probability of release of the Weibel-Palade body (WPB) is determined by a balance favoring exocytosis and the fractional occupancy of WPB-Rab27A by the effector proteins Slp4-a and MyRIP. [35] Similarly, in our study, MVB secretion as exosomes might have been enhanced by an imbalance in intracellular MVB trafficking and docking, which is regulated by RAB27A. We hypothesized that when RAB27A was overexpressed, the probability of exosomes and miRNAs being secreted from cancer cells was higher, and the metastatic potential was more significant than when RAB27A was knocked down. [23, 35] Based on electron microscopic analysis, the number of ILVs per MVBs did not show a significant increase in the RAB27A overexpressing cells. However, ILVs and MVBs per cell were increased, indicating that RAB27A OE may increase exosome secretion.

Next, we quantitatively analyzed the particle counts of secreted exosomes in RAB27A overexpressing RCC compared with those in the control group using Exoview analysis. We created a mature miRNA beacon to analyze the secreted miRNA particle count and to evaluate their sorting and secretion within exosomes. In addition, we identified the most significant miRNAs sorted in exosomes by applying previous research on exomotifs and found them downregulated in RAB27A OE RCC using RNA sequencing. Through PPI and ceRNA network analysis, we found MYCN to be the most significant hub of metastatic RCC, and miR-127-3p and RAB27A as therapeutic targets. MYCN protein levels were higher, and its mRNA levels lower, in RAB27A-OE carcinoma cells than in the control ACHN cells (Additional file 1). This MYCN overexpression without amplification might be due to the post-transcriptional regulation. These finding suggest that, in RCC cells, the RAB27A-OE-induced increase in exosome secretion caused MYCN protein levels to increase, thus increasing their metastatic potential. Exosome sorting of this RNA cargo, miR-127-3p, has been theoretically demonstrated to have an exomotif and was experimentally proven in this study to be downregulated in RAB27A overexpressing cells detected by RNA sequencing. Finally, the migratory activity of the RCC cells was evaluated by incubating them in different cell-derived exosome media. The same numbers of RCC cells were exposed to these different media; therefore, a difference in the observed migration rate would indicate that the cellular uptake of exosomes and miRNAs contained in the medium was affected. Cancer cells treated with control cell-derived exosome media showed more migration than to RAB27A overexpressing cell-derived exosome media. Comparing this result to a previous study conducted in 2007, it is possible that among the secreted miRNAs within the exosome media, critical tumor suppressor genes that determine cancer metastatic potential might have been taken up by recipient cells.[3, 25]

Of the 28 representative miRNAs in the RAB27 OE group, we identified 6 candidate miRNAs that were probably sorted into exosomes by applying previous results on exosome RNA cargo sorting. Five genes (miR-127-3p, miR-136-3p, miR-409-3p, miR-376a-5p, and miR-134-5p) had exomotif sequences [30], and miR-323a-3p was found to be related to Ago2 [29] (Figs. 6c and 6d). We identified six candidate miRNAs that were likely sorted into exosomes using RNA sequencing, ceRNA network analysis, and protein–protein interactions with gene ontology term enrichment analysis.

We selected miR-127-3p for further evaluation for several reasons: 1) Among the well-known tumor-suppressor genes that suppress Bcl-6 in many types of cancer, it is one of the most important miRNAs; 2) It has EXOmotif sequences that are may be sorted into exosome cargo; and 3) Among the genes downregulated in RAB27A-OE RCC cells (detected by miRNA sequencing), it had one of the highest fold changes (− 339.420703) (p = 2.28705E-13).

**Fig. 12:**
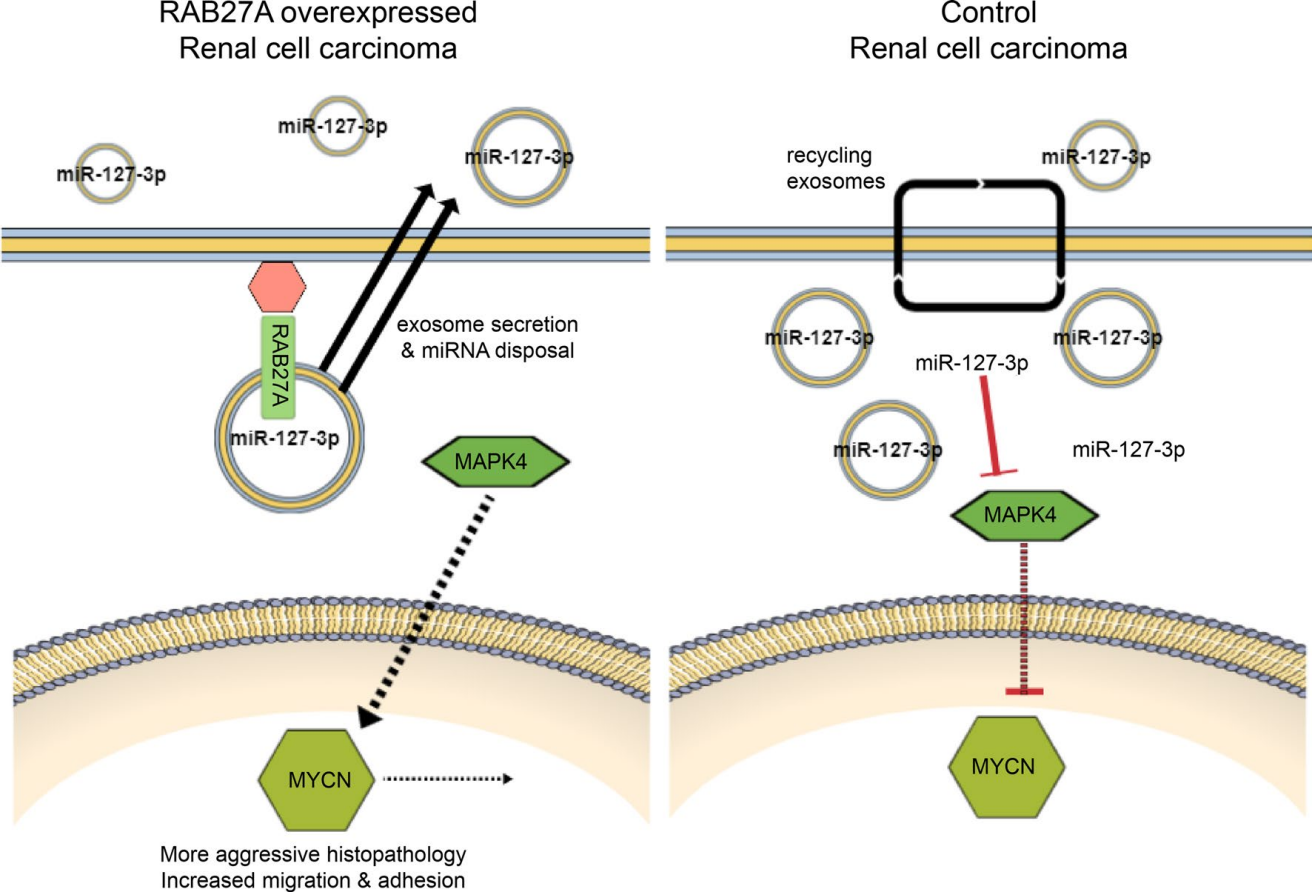
Role of miR-127-3p in oncogenic regulation. By disposing of miR-127-3p through exosome secretion of RAB27A overexpressing cells, which may not inhibit MAPK pathway to gain cancer metastatic potential, including more aggressive histopathology, and increase in migration and adhesion by activating MYCN transcription factor. In contrast, in control cells, the recycling of exosomes is maintained, and the number of miR-127-3p in cancer cells is balanced to increase post-transcriptional change and inhibiting signaling pathways, including MAPK and MYCN

Via Exoview analysis, we used miR-127-3p as a fluorescence RNA probe to identify secretion of this gene as an exosomes cargo. ceRNA network analysis revealed that it interacted strongly with MAPK4 (Fig. 8), a kinase that belongs to three major downstream signaling cascades of the RAS oncoprotein. miR-127 is one of the tumor suppressor genes located on chromosome 14q32.31, encodes a protein that suppresses Bcl-6. Several signaling pathways regulate BCL6 expression at the transcriptional and post-transcriptional levels; these include the B-cell receptor (BCR) signaling, ATM-promoted DNA damage, and CD40 signaling pathways. [36, 37] When BCR activation is induced by antigen stimulation, MAPK-mediated phosphorylation of BCL-6 may lead to the degradation of BCL6 via ubiquitination. [36] In this study, we identified MAPK4 interacted strongly with miR-127-3p. We hypothesized that BCL-6 by miR-127-3p may be linked to a signaling pathway involving MAPK4 signaling by BCR. In RAB27A overexpressing cells, displacement of miR-127-3p via exosome secretion may not inhibit the MAPK pathway to gain cancer metastatic potential by activating transcription factors such as the MYC oncogene (Fig. 12).

The MYC pathway is known to be activated in most human RCC and thus has been an interesting target for developing RCC models in mice. Myc expression was driven by the kidney-specific GGT promoter coupled to the tetracycline transactivating gene (tTA) and the induction of Myc resulted in the fast development of RCC [38]. In this study, through protein–protein interactions of 167 protein-coding RNAs of RAB27A overexpressing cells that are likely to be involved in cancer metastatic potential, we identified MYCN as one of the most significant hubs that interact with various other genes. Based on the findings of our in vitro study, we hypothesize that the metastatic potential of in RAB27A overexpressing cells, reflected by cancer cell phenotypic properties as migration, proliferation, cell adhesion, and cell differentiation, might be accelerated via MYCN-induced activation of the MAPK signaling pathway, possibly due to a decrease in post-transcriptional changes owing to exosomal secretion of the tumor suppressor gene miR-127-3p. (Fig. 12).

Signaling pathways including BCR, MAPK, and MYC, may affect RCC metastasis. MYCN amplification has been associated with undifferentiated, aggressive characteristics of neuroblastoma in many studies. Mutations in the RAS–MAPK pathway frequently occur in recurrent neuroblastomas, suggesting that activation of this pathway is related to a more aggressive phenotypic change. [39, 40] MYC inhibition has been considered a therapeutic option for malignant cancers. However, the non-therapeutic protein structure obscures the direct target-MYC inhibitors. [41] The clinical approaches for blocking this class of molecules of the transcription factor MYCN remains to be determined. One of the most direct means of silencing these molecules is to disrupt Myc synthesis via RNA interference. Although effective inhibition of MYC using siRNA has been demonstrated in several in vitro experiments, this method is not widely used in clinical trials because of difficulties related to its delivery. [42, 43] In this respect, since we confirmed that MYCN exists as the most potent mutation in kidney cancer [38], we aimed to present a therapeutic option with the same effects as RNA interference, by using exosomes to carry a cargo of this microRNA. Stabilizing and preventing the secretion of exosomes may help miRNAs to regulate Myc synthesis.

Currently, exosome-related cancer treatment studies are developing drugs that control the tumor microenvironment by targeting the original cells or via exosome-based drug-delivery. Cell-to-cell communication affects tumor development and metastasis by transferring exosomes from specific cancer to surrounding cancer cells in the tumor microenvironment. [3, 19, 44–47] The inappropriate secretion of exosomal miRNAs may cause significant changes in biological pathways and induce cancers and other developmental diseases. [44–47] The therapeutic potential of miRNA-containing exosomes has been elucidated. [44, 48] However, until recently, only a few studies have addressed the cellular or molecular mechanisms determining exosome biogenesis, sorting, secretion, and uptake. [8, 19, 44, 47, 48] It remains to be determined whether cellular uptake of exosomes occurs via micropinocytosis, macropinocytosis, or receptor-mediated pathways (the main route) and whether cargo delivery is possible through this process [3, 19]. In this study, through next-generation sequencing and in vitro experiments, we demonstrated that several miRNAs, including miR-127-3p, may have been sorted into exosomes and secreted extracellularly by RAB27A overexpression to accelerate cancer cell metastasis. Here, we present a novel RCC therapy that inhibits MYCN by stabilizing RAB27A and preventing exosome secretion and miR-127-3p disposal. Our finding connects those of previous studies on the exosome sorting mechanism of the EXOmotif and the disposal of tumor suppressor miRNA by RAB27A, which could affect cancer cell metastasis. [26, 29, 30] Future research into specific miRNAs related to metastasis, using RAB27A-related exosome secretion, will substantially alter the treatment of many cancers.

In this study, we identified the relationship between miR-127-3p and MAPK4 in RAB27A overexpressing cell and their relationship with the signature hub MYCN (the main subject of the study) using RNA sequencing data bioinformatics analysis. Nonetheless, these findings are limited in that we have yet to prove RAB27A-OE cell metastasis and the association between RAB27A-OE cells and miR-127-3p through animal experimentation. We expect further studies to reveal the biological mechanisms of action of RAB27A.

## Conclusion

We have demonstrated that the tumor suppressor gene miR-127-3p, secreted by RAB27A-OE RCC cells, underwent cargo sorting and secretion by exosomes; this induced MYCN expression and accelerated metastasis. Future exosome research on cancer treatment in patients with metastatic RCC may achieve better stabilization of RAB27A and more effective prevention of exosome secretion of miR-127-3p.

### Supplementary Information

**Additional file 1.** Additional figures.

## Data Availability

The datasets used and analyzed in the current study are available from the corresponding author on reasonable request.
