# Supplementary material for: Exosome-mediated secretion of miR-127-3p regulated by RAB27A accelerates metastasis in renal cell carcinoma
Source: Cancer Cell Int. 2024 Apr 29;24:153. doi: 10.1186/s12935-024-03334-0 (PMC11057152; doi:10.1186/s12935-024-03334-0)
Supplement: Supplementary file 1 — Additional file 1. Additional figures. [file 12935_2024_3334_MOESM1_ESM.pdf]

SUPPLEMENTAL INFORMATION

MANUSCRIPT TITLE: Exosome-mediated secretion of miR-127-3p regulated by RAB27A accelerates metastasis in renal cell carcinoma

Dae Hyun Song[1,2,3], Jong Sil Lee[2,3,4], Jeong-Hee Lee[2,3,4], Dong Chul Kim[2,3,4], Jung Wook Yang[2,3,4], Min Hye Kim[4], Ji Min Na[4], Hyun-kyung Cho[2,5], Jiyun Yoo[6], Hyo Jung An[1,2,3]

[1]Department of Pathology, Gyeongsang National University Changwon Hospital, Changwon, Republic of Korea, [2]Institute of Medical Sciences, Gyeongsang National University, Jinju, Republic of Korea, [3]Department of Pathology, Gyeongsang National University School of Medicine, Jinju, Republic of Korea, [4]Department of Pathology, Gyeongsang National University Hospital, Jinju, Republic of Korea, [5]Department of Ophthalmology, Gyeongsang National University Changwon Hospital, Gyeongsang National University, School of Medicine, Changwon, Republic of Korea, [6]Division of Applied Life Science (BK21 Plus) and Research Institute of Life Sciences, Gyeongsang National University, Jinju, Republic of Korea

SUPPLEMENTAL DATASET 1

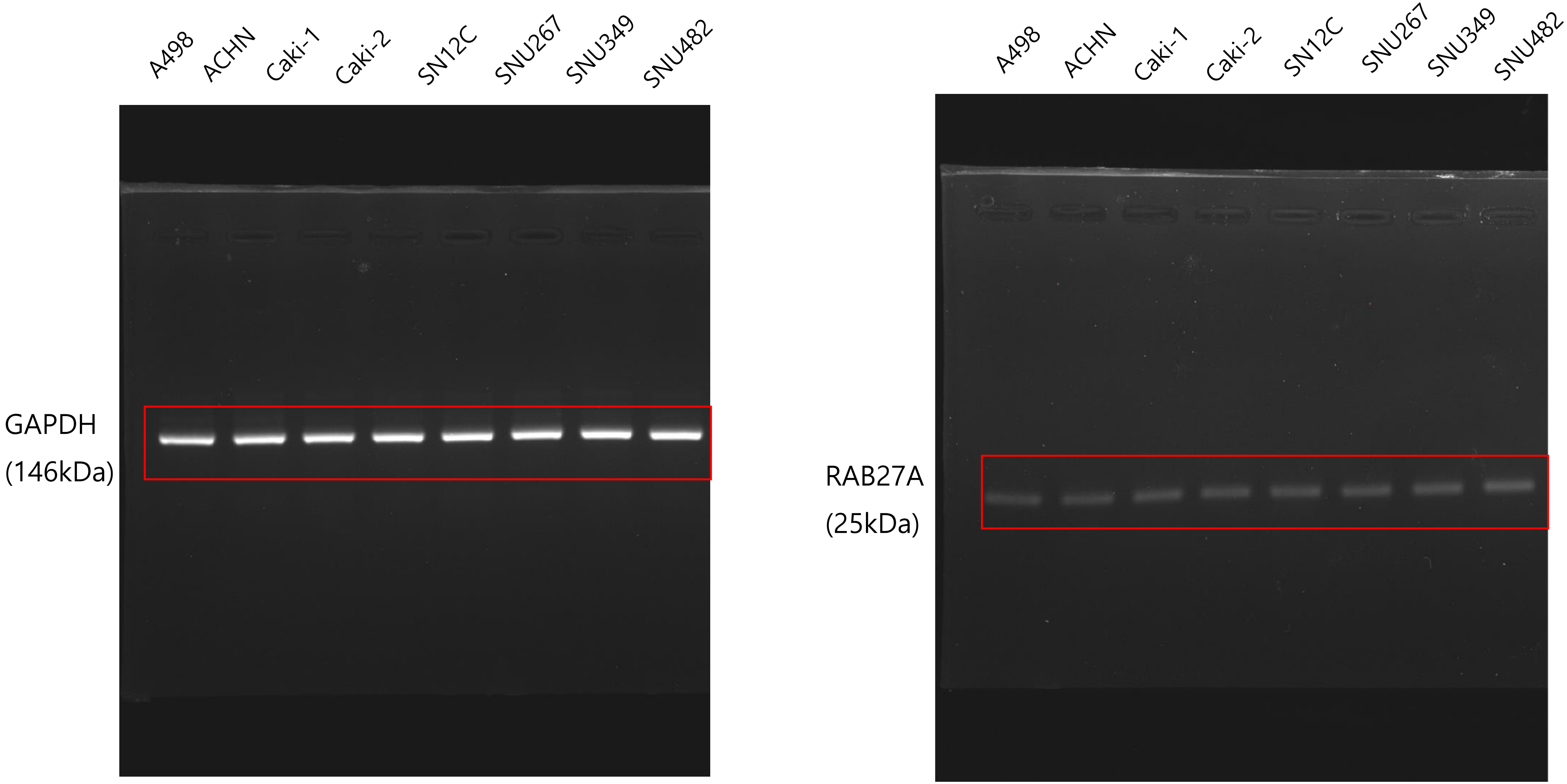

Figure S1.Original raw semi-qPCR data of agarose gel electrophoresis for Figure 1A.

SUPPLEMENTAL DATASET 2

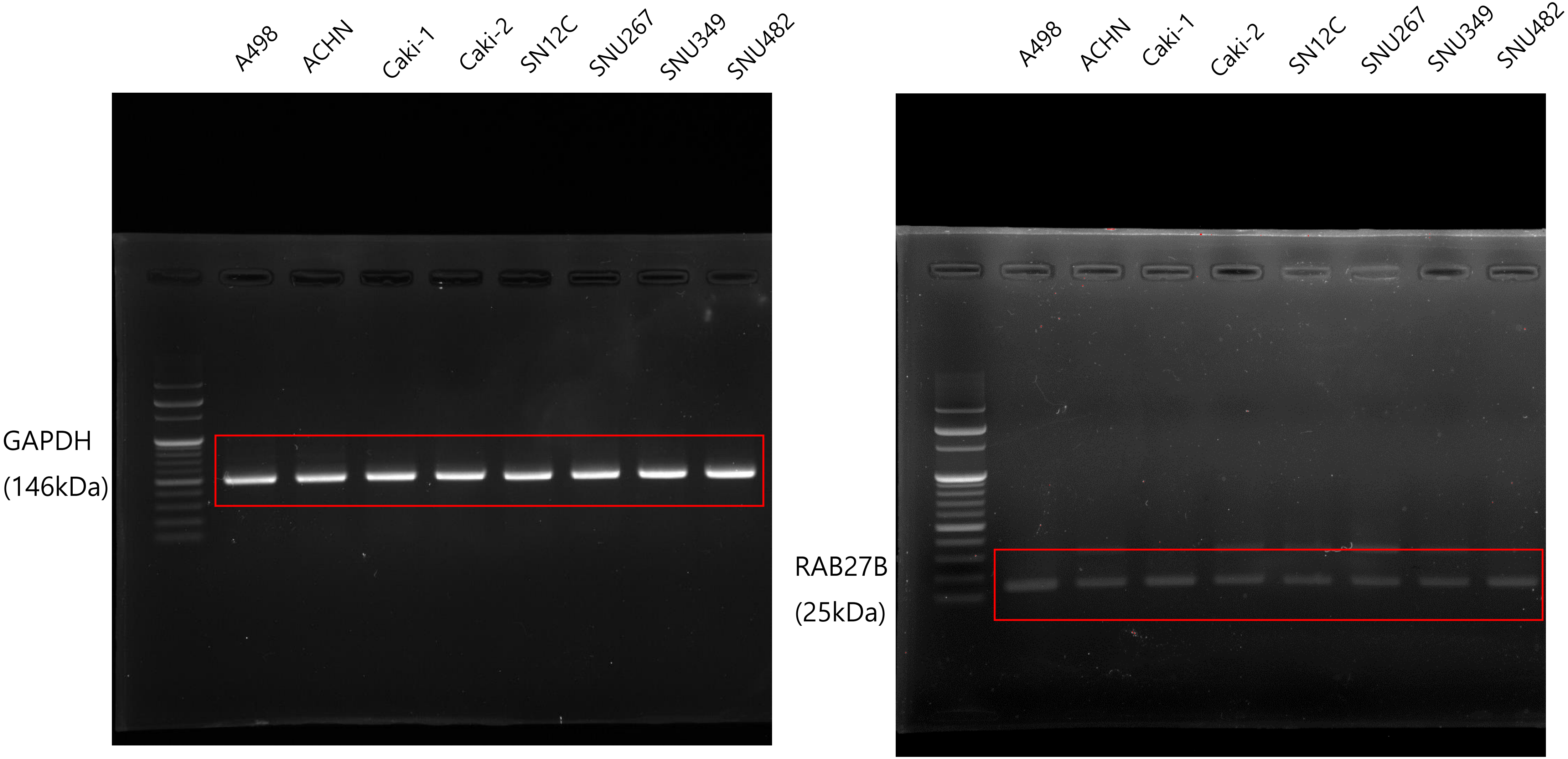

Figure S2.Original raw semi-qPCR data of agarose gel electrophoresis for Figure 1B.

SUPPLEMENTAL DATASET 3

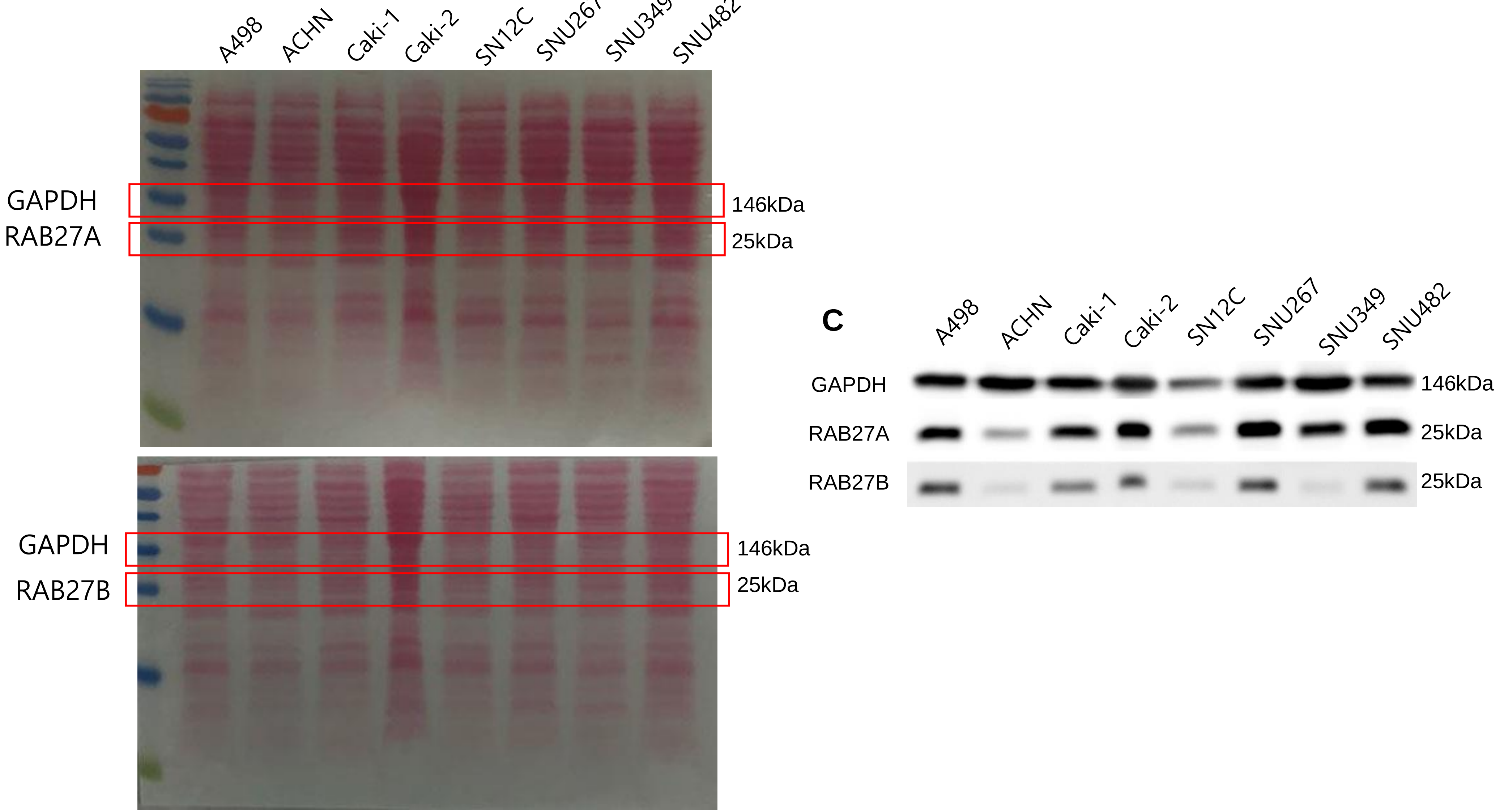

Figure S3. Original raw Western-blot data for Figure 1C.

SUPPLEMENTAL DATASET 4

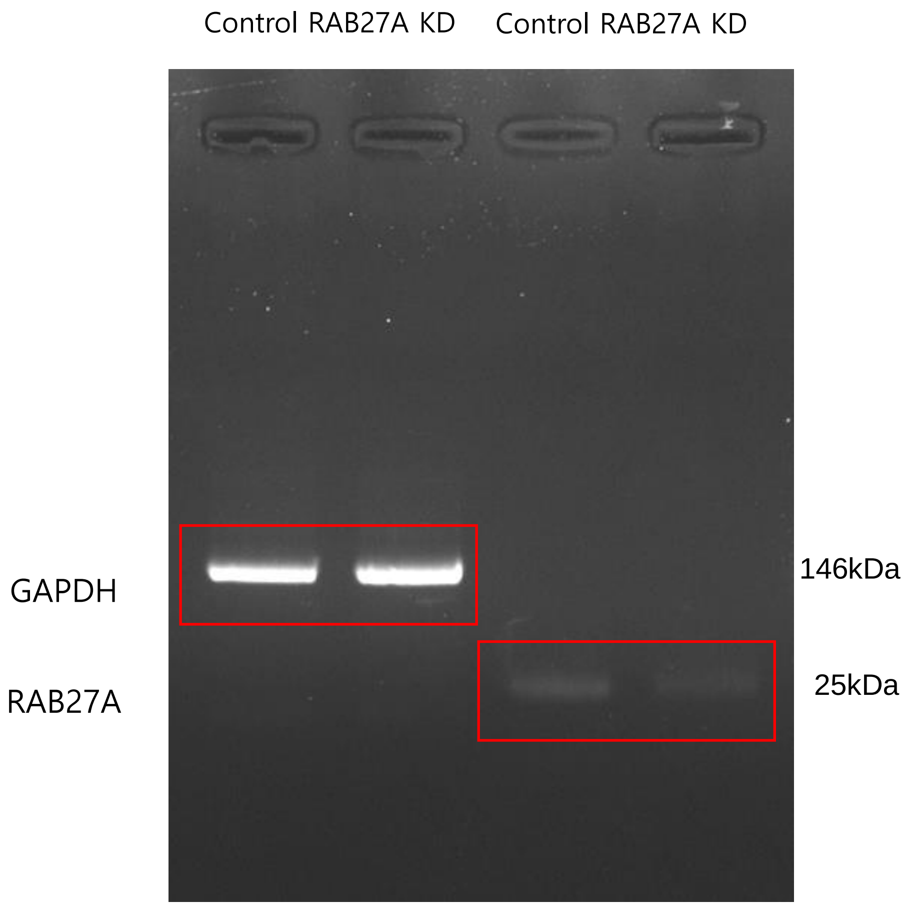

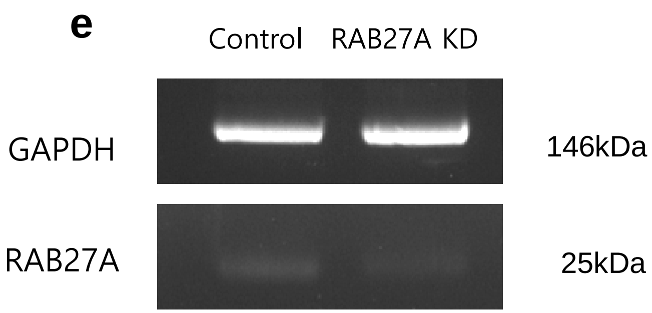

Figure S4. Original raw semi-qPCR data for Figure 1E.

SUPPLEMENTAL DATASET 5

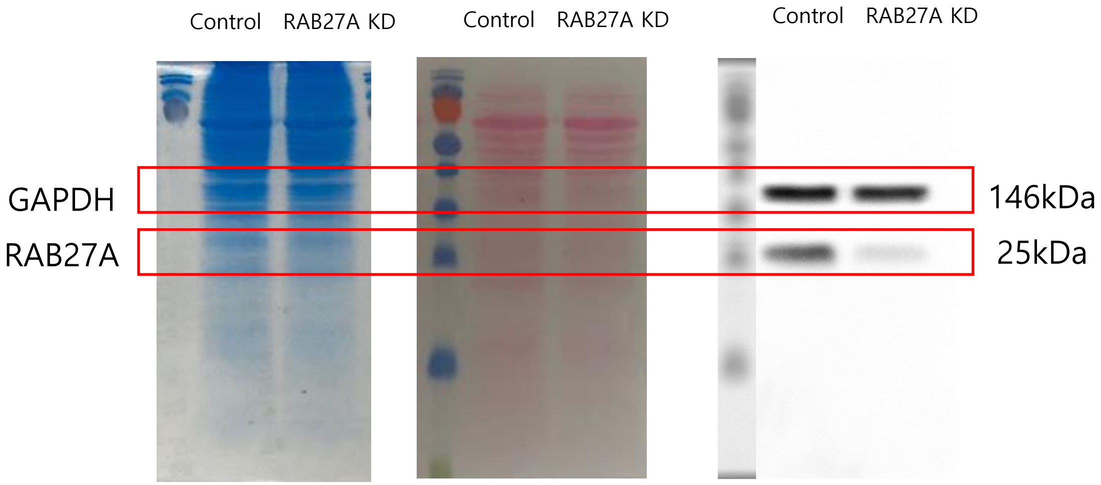

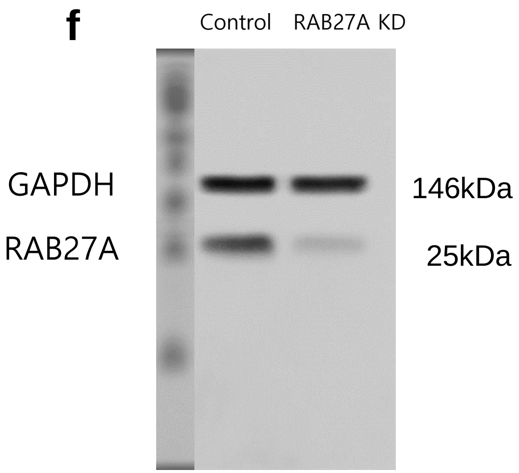

Figure S5. Original raw Western-blot data for Figure 1F.

SUPPLEMENTAL DATASET 6

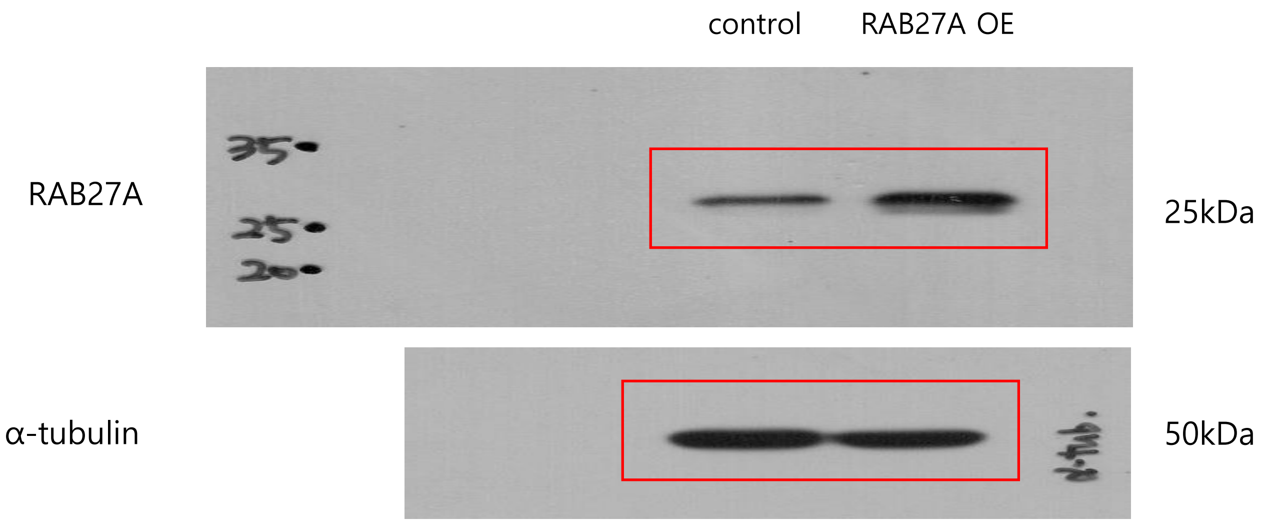

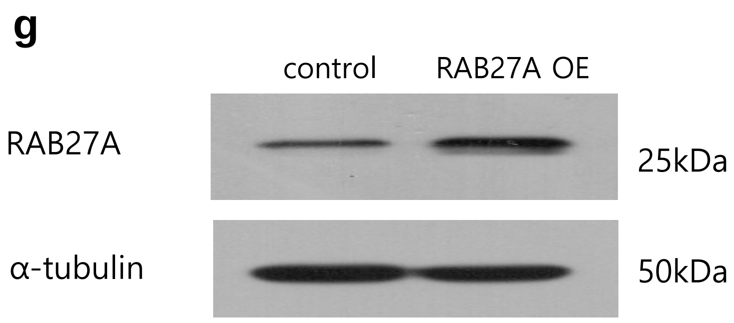

Figure S6. Original data of Western-blot for Figure 1G.

SUPPLEMENTAL DATASET 7

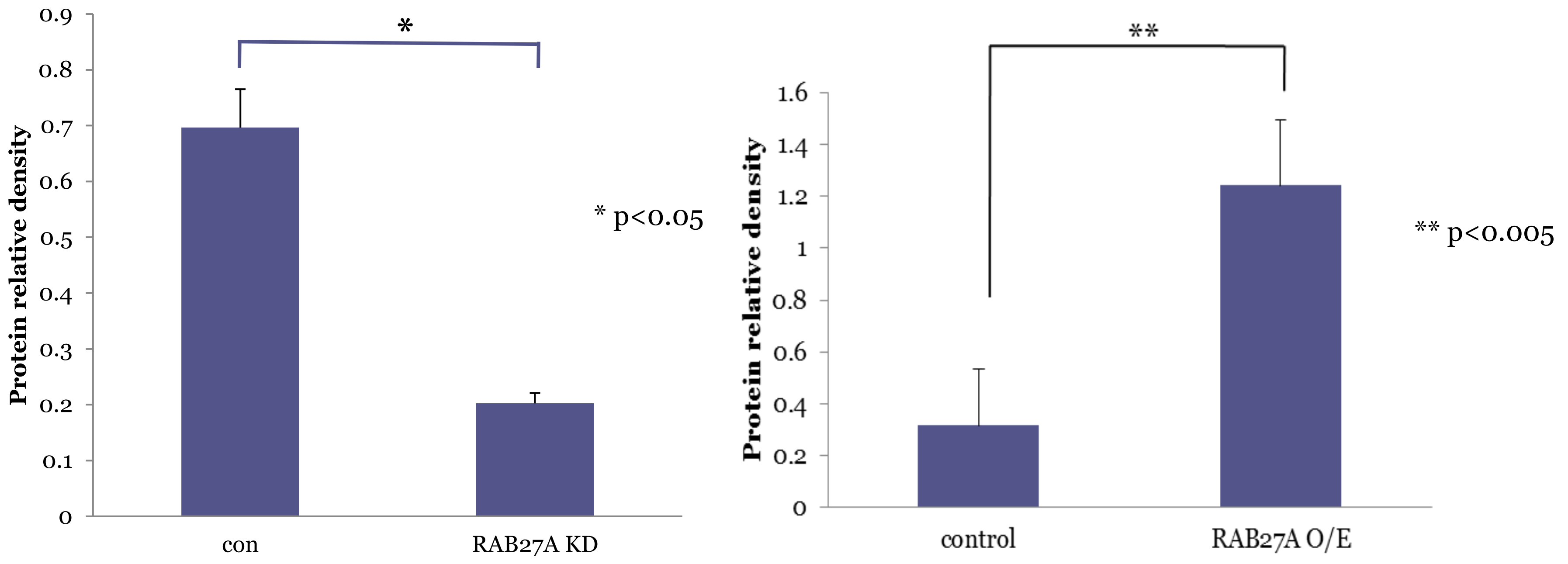

Figure S7. Relative Protein density for Figure 1F and Figure 1G.

SUPPLEMENTAL DATASET 8

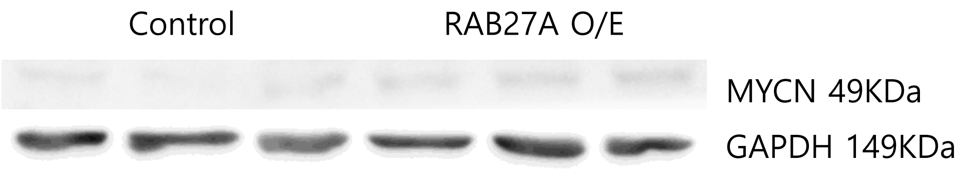

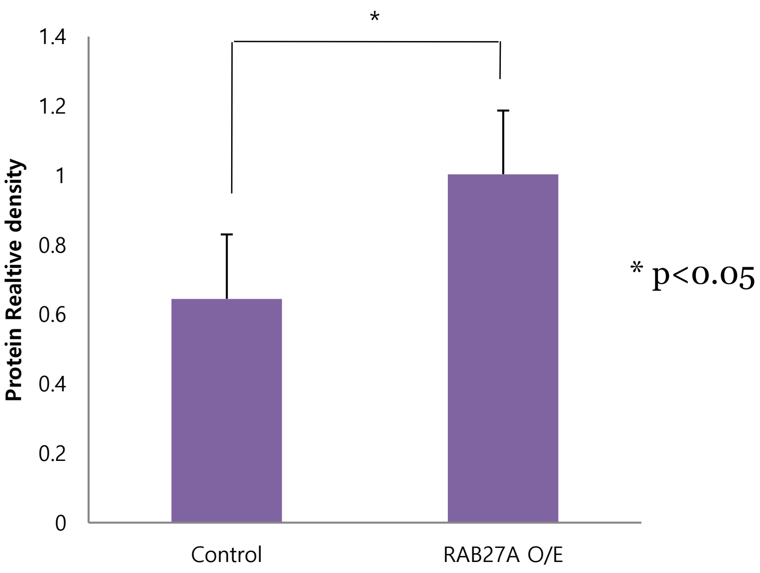

Figure S8. Relative Protein density for MYCN in RAB27A OE renal cell carcinoma cells.

SUPPLEMENTAL DATASET 9

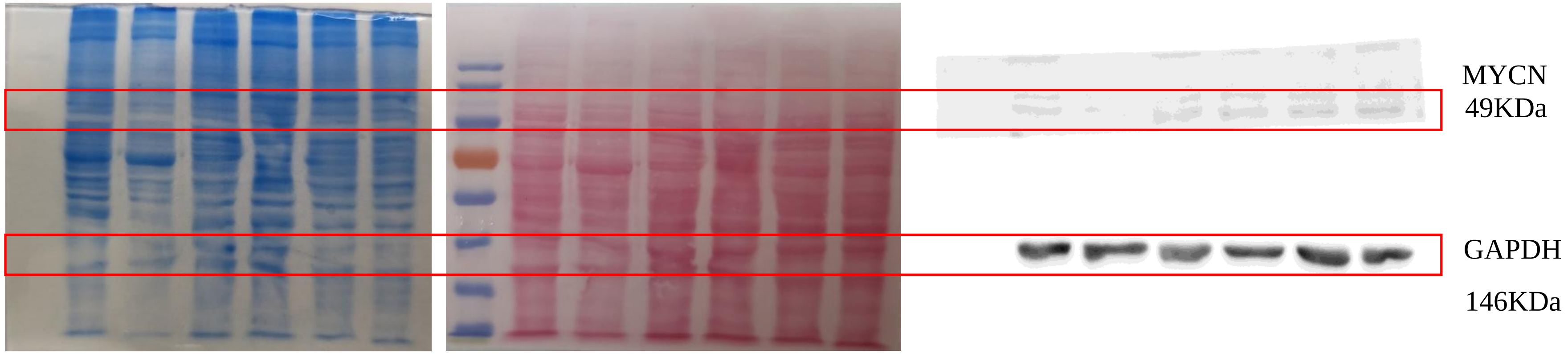

Figure S9. Original raw Western-blot data for figure S8 (MYCN protein expression in RAB27A OE renal cell carcinoma cells)

SUPPLEMENTAL DATASET 10

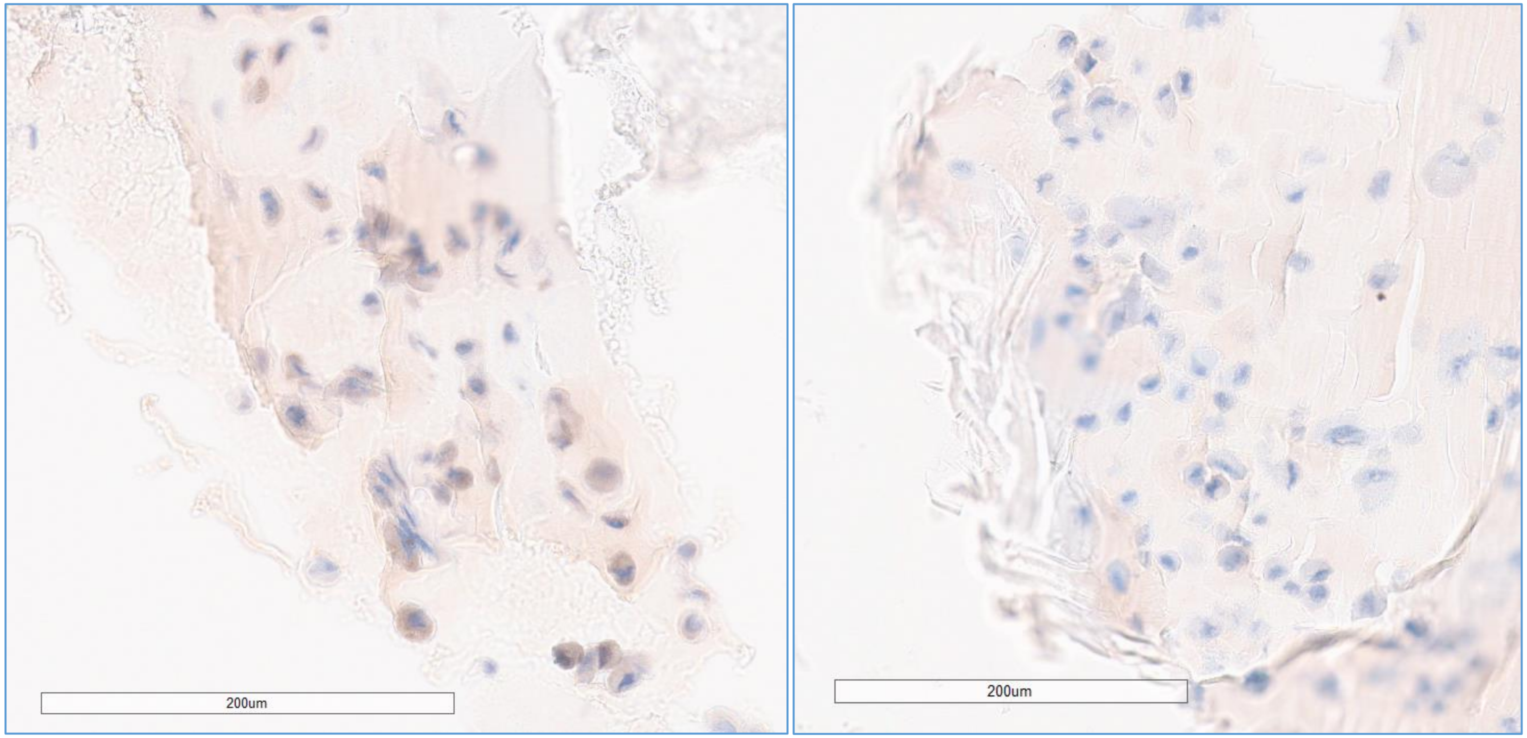

Figure S10. Immunohistochemical staining of MYCN on the cell blocks of RAB27A OE cells (left) and control ACHN cells. The higher cytoplasmic and membranous MYCN expression was identified in the RAB27A OE cells on the left. On the other hand, weaker and lower cytoplasmic MYCN expression is identified in the ACHN cells on the right.

SUPPLEMENTAL DATASET 11

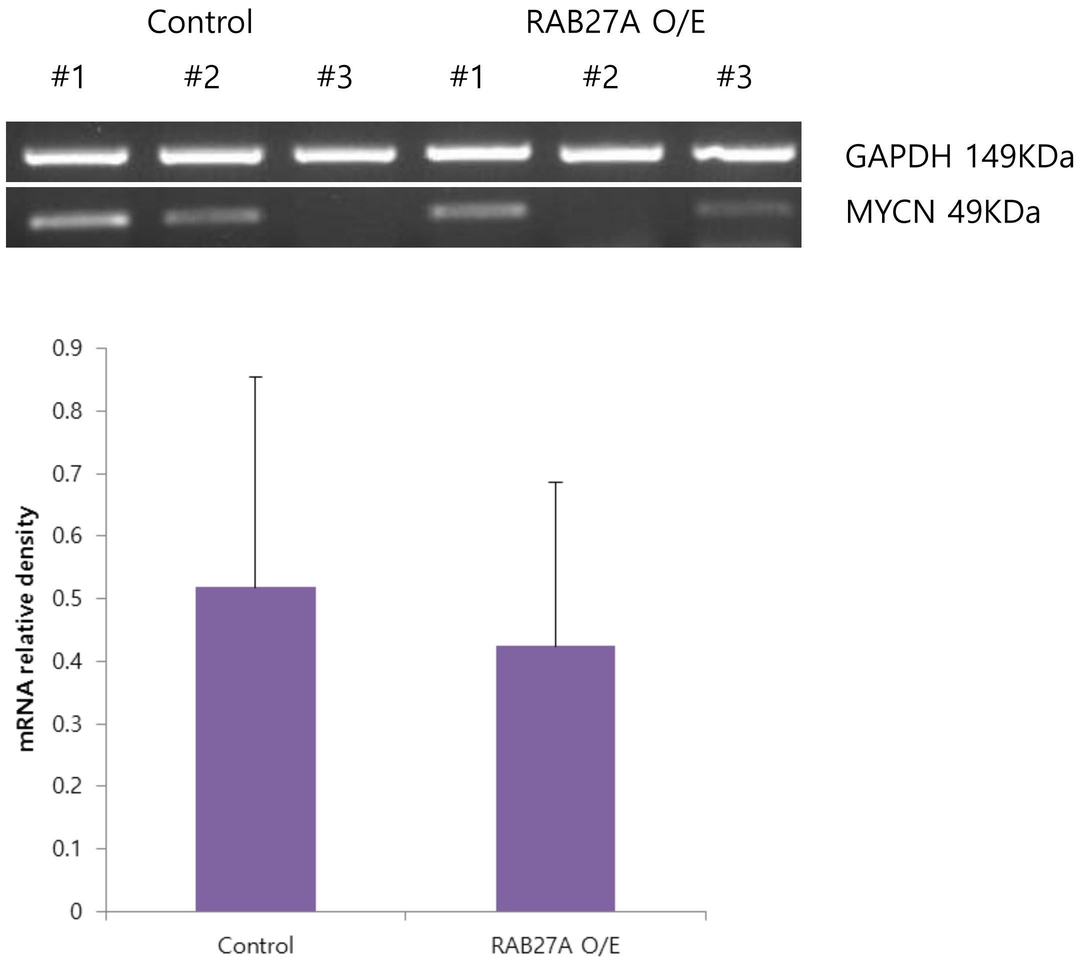

Figure S11. Relative mRNA density for MYCN in RAB27A OE renal cell carcinoma cells.

SUPPLEMENTAL DATASET 12

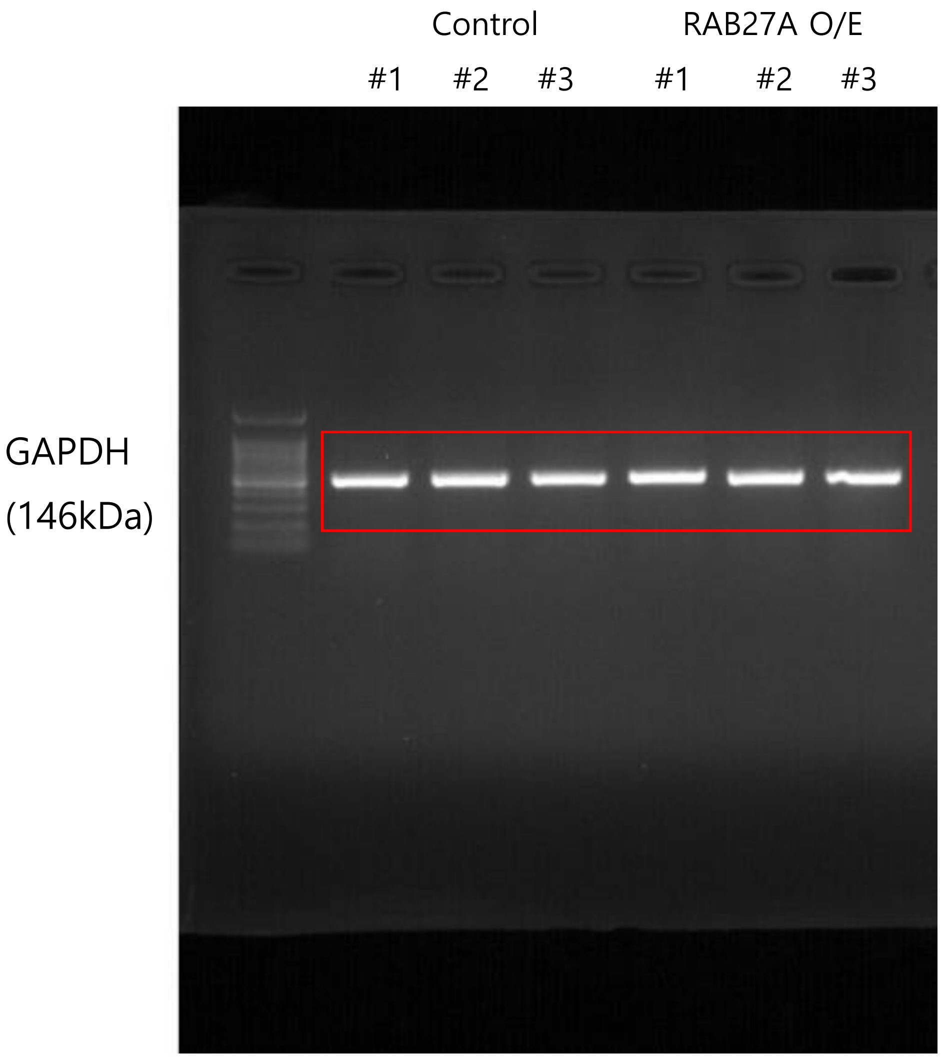

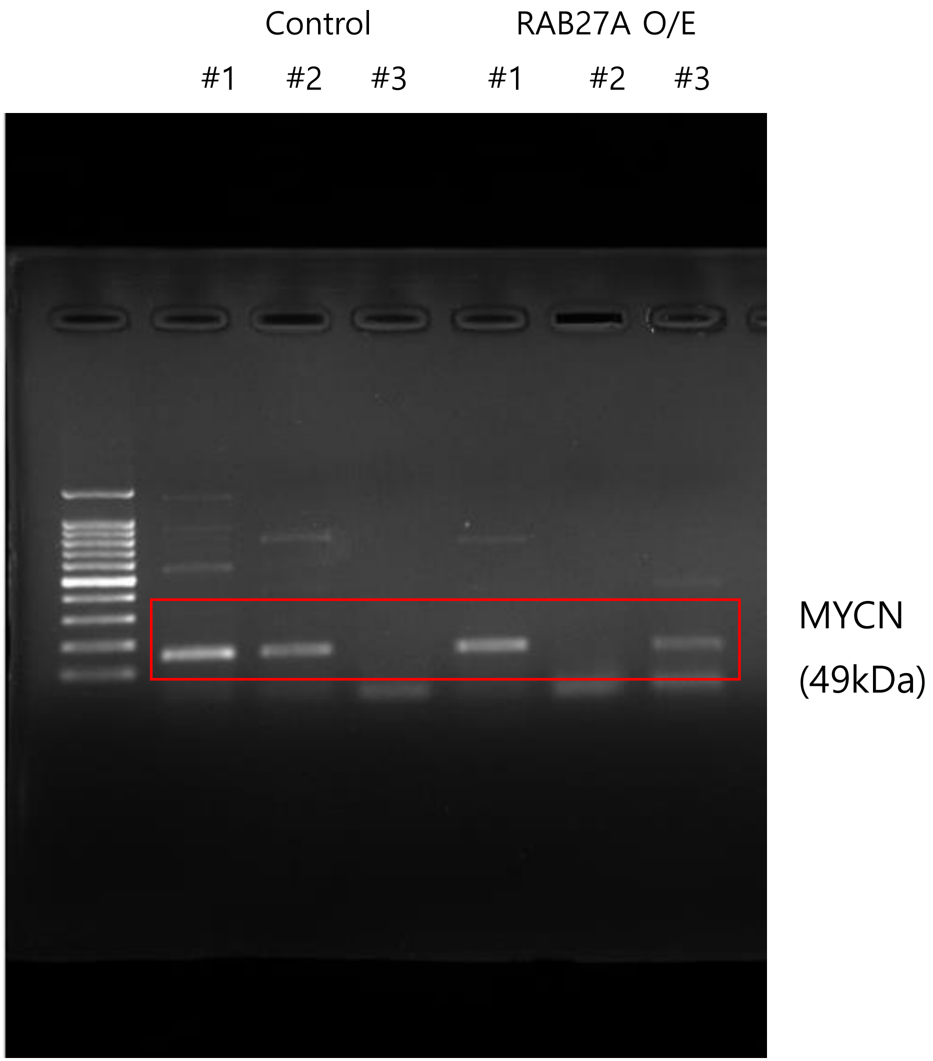

S12.Original raw semi-qPCR data of agarose gel electrophoresis for S11.
